# From density functional theory to machine learning predictive models for electrical properties of spinel oxides

**DOI:** 10.1038/s41598-024-62788-4

**Published:** 2024-05-27

**Authors:** Yuval Elbaz, Maytal Caspary Toroker

**Affiliations:** 1 Department of Materials Science and Engineering, Technion – Israel Institute of Technology, 3600003 Haifa, Israel; 2 The Nancy and Stephen Grand Technion Energy Program, Technion - Israel Institute of Technology, 3600003 Haifa, Israel

**Keywords:** Computational methods, Electronic structure

## Abstract

This work focuses on predicting and characterizing the electronic conductivity of spinel oxides, which are promising materials for energy storage devices and for the oxygen evolution and oxygen reduction reactions due to their attractive properties and abundance of transition metals that can act as active sites for catalysis. To this end, a new database was developed from first principles, including band structure and conductivity properties of spinel oxides, and machine learning algorithms were trained on this database to predict electronic conductivity and band gaps based solely on the compositions. The models developed in this study are scaled from the quantum level up to a continuum conductivity model. The relatively small database used in this study allowed for accurate predictions of band gap and conductivity. By altering the composition of spinel oxides, the model was able to predict high conductivity for spinels with high nickel content and to match experimental trends for manganese cobalt spinels. The ability to predict material properties is especially important in energy conversion devices such as batteries and supercapacitors where redox reactions take place.

## Introduction

The increasing demand for renewable energy has led to a significant focus on energy storage devices, such as batteries and supercapacitors. Improving the electrochemical efficiency of these devices is crucial to their development, and spinel materials are commonly used as both the anode and cathode of Li-ion-based storage devices^1–3^. The structural and compositional versatility of spinels allows for various engineering paths to improve device performance by altering the stoichiometry or elements of the spinel oxide to achieve higher or lower electrical conductivity^4^. Predicting these properties before experimentation is of great interest and can be achieved through simulations and machine learning algorithms.

Ternary metal oxides are particularly attractive due to their low cost, high theoretical specific capacitance, multiple oxidation states, and environmental friendliness^5^. Among them, ternary metal oxides of spinel structure are represented by the general formula AB_2_O_4_, where A and B are transition metals. The normal spinel has a cubic unit cell consisting of eight FCC cells made up of oxygen atoms, creating an array of octahedral and tetrahedral sites for cations. The divalent A metals occupy 1/8th of the tetrahedral sites, while trivalent B metals occupy 1/2 of the octahedral sites, resulting in eight tetrahedral and sixteen octahedral sites in a unit cell. Inverse spinels have the same structure, except for ions from the tetrahedral sites and half of the ions from the octahedral sites swapping places. The chemical formula can be expanded to (A_y_B_1−y_)[A_x_B_2−x_]O_4_, where A and B metals can occupy tetrahedral (T_d_) or octahedral sites [O_h_], and 0 < y < 1 and 0 < x < 2. The complexity of this material type's composition and its impact on electrical properties has traditionally made it difficult to discover and optimize materials. However, data-driven approaches in material science offer new avenues for identifying promising compositions and accelerating the search for novel materials with desired electrical properties.

Data-driven methods are now widely employed in various technological fields, including computational materials science. Machine learning (ML) is a mathematical model that improves with data amount, and its uses include regression, classification, clustering, anomaly detection, and dimensionality reduction. Integrating ML with ab-initio approaches can significantly accelerate materials discovery and property calculations by predicting various materials’ properties based on their geometry and elements. For example, ML methods have been applied to predict various material properties from their geometries. For instance, the dielectric breakdown strength of perovskites was predicted with a ML model based on a phenomenological theory^6^. The calculations of Green functions for the electronic transport of 1D nanostructures were accelerated with ML approaches^7^. A ML algorithm reduced the computational cost of charge transport prediction in DNA molecules by an orders of magnitude^8^. A combination of different ML algorithms helped find a correlation between the structure and the electron transfer property of a nanoparticle^9^. Another recent example is the usage of ML algorithms to parameterize the tight-binding models of defect structures in novel materials^10^. These examples illustrate how ML can predict material properties from basic knowledge of the material, such as its geometry and elements, which can accelerate the discovery of new materials and heuristic models.

To address the challenge of exploring the vast array of spinel oxide compositions and their resulting conductivities, it is not feasible to synthesize or simulate all possible combinations. Instead, we suggest to generate a dataset of simulated materials to cover a representative portion of the total combinations. This dataset can then be used to train ML models that find the function linking composition to conductivity or band gap. Once trained, these models can predict the conductivity or band gap of all other compositions that were not calculated or synthesized. This approach can accelerate the discovery of new spinel materials for electrochemical electrodes.

Gathering data to provide input for machine learning projects is a crucial and often arduous initial stage. In recent years, substantial databases for materials science and chemistry have emerged, featuring a combination of experimental and computational results^11–14^. These comprehensive datasets are now available for users to explore and apply for data science purposes. Despite the availability of numerous databases, we were unable to locate a suitable option that met our requirements—namely, samples of various spinel oxides, not limited to their typical structures, and their corresponding conductivity values. As a result, we opted to construct our own database by conducting DFT calculations to determine electronic structures, and electronic current simulations utilizing non-equilibrium Green’s function (NEGF) and Landauer methodologies.

In this paper, we present a novel scheme for making conductivity predictions in spinel oxide materials and demonstrate its effectiveness through its application to a range of spinel oxide systems. To this end, we are unaware of any work with electrical conductivity calculations which covers a diverse range of spinel oxides in a systematic way as achieved in this study. The scheme takes advantage of various methods, we first used density functional theory (DFT) to build a dataset of these materials and calculated their band structures. We fitted the band structures to tight-binding Hamiltonians and used these Hamiltonians as an input to current calculations following Landauer–Büttiker formalism. By this, we achieved a dataset of 190 different ternary spinel oxides and their associated current flow under 1 V bias. The various calculated spinel oxides in this dataset contain Fe, Mn, Co, and Ni as their A, and B transition metals at different stoichiometries. This dataset is then used to train ML models to predict the current of out-of-set samples based on the composition of the sample. In addition, more ML models were trained to predict the current based on the bandwidths of the bands of the material and by this saving the time of doing current calculations. Furthermore, a model to predict the band gap of the material based on its composition was trained as well.

## Results and discussion

In the first section of the results, we analyze the database of spinel oxides that was used to train the prediction models. The database is based on the DFT, tight-binding band fitting, and NEGF calculations. After that, we present the results of three different types of predictors: composition → current, bandwidth → current, and composition → band-gap.

### Building the dataset and database properties

The project of prediction models began by creating a database of spinel oxides. This was achieved by performing DFT calculations on different compositions of spinel oxides. Once the relaxed geometries of these compositions were determined, then their band structures along high-symmetry k-points were computed. The band structures were then fitted to tight-binding Hamiltonians with the procedure described in the methods section. The current at 1 V bias for each composition was then calculated using NEGF and Landauer formalism.

The database contains calculations for 190 different ternary spinel oxides. Each with different x, y, A, B of the formula A_y_B_y−8_A_16−x_B_x_O_32_. All six combinations of Fe, Ni, Mn, and Co as the A, B elements were considered. The magnetic moments of each ion were evaluated to find anomalies or inconsistencies. When such anomalies were detected we re-run the DFT optimization with a new initial magnetic moment in order to get a reasonable value of the converged magnetic moment (magnetic moments results can be found in the supplementary information, Figure S10-S15).

**Figure 1:**
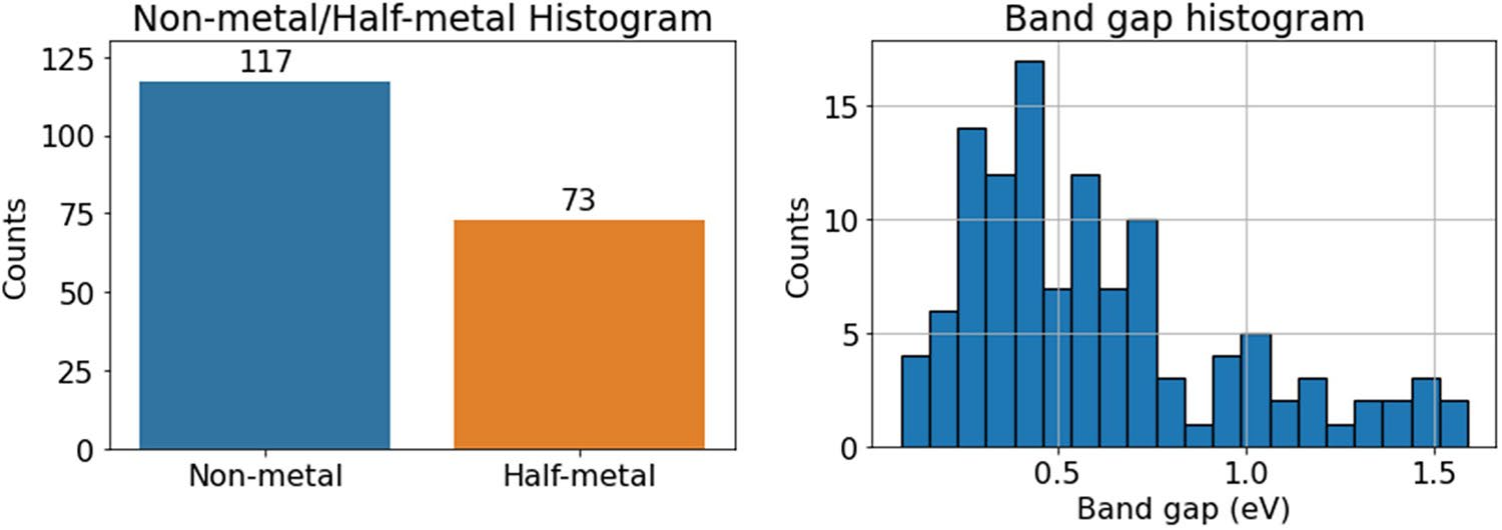
DFT calculations results of the band gap for spinel oxides. Left: Half-metal/semiconductor histogram. Right: Band gap histogram.

After geometric relaxation was done, the band structure of each composition was calculated with DFT. From these bands, we first explore the band gap property. Figure 1 shows the number of zero and non-zero band gaps. We constructed a histogram to analyze the dataset, as shown in Fig. 1 (left). The histogram displays the number of non-metal/semiconductor compounds and half-metallic compounds. A half-metal is a solid that has a band gap for one spin direction and no band gap for the other spin. Our analysis revealed that 73 compositions exhibited half-metallic behavior, while 117 behaved as semiconductors with a band gap for both spin directions. Additionally, the band gap of the semiconductor materials was defined as the lowest energy gap among the gaps produced by spin up and down. Figure 1 (right) illustrates the distribution of different band gap values, with a minimum of 0.083 eV and a maximum of 1.59 eV. These results were utilized to train a model that predicts the band gap of a spinel based on its stoichiometry, as detailed later in the results.

In the following step, we applied the method outlined in the methods section to fit all band structures to a tight-binding Hamiltonians in order to perform efficient NEGF calculations. To assess the accuracy of the tight-binding Hamiltonians, we compared the band structure generated by the Hamiltonians to the DFT band structure. We calculated the absolute difference between the eigenvalues of the TB and DFT and took the mean value as the error (mean absolute error, MAE). Figure S1 displays histograms of the MAE for spin-up and spin-down band structures. We found that the majority of TB Hamiltonians produced MAE of 0.02 eV or less, with spin-down band structures being slightly more accurate. This is likely because the minority spins in spinels produce narrow bands on average, which are easier fitted to a tight-binding Hamiltonian compared to wider bands. The variance of the MAE for each sample was approximately 0.02 eV or less, except for only two cases where it reached 0.06 eV that had wide bands and were difficult to fit so they are excluded from the figure. These results demonstrate that the TB Hamiltonians provide an excellent fit to the DFT band structures, enabling the accurate calculation of conductivity properties.

We calculated the current under a bias of 1 V using NEGF with the TB Hamiltonians of each sample. The obtained current values were utilized for training machine learning (ML) models for current prediction. The distribution of these current values is depicted in Figure S2. The mean value of all current results is 149.7 μA, and the standard deviation is 108.5 μA, with a maximum value of 755.4 μA and a minimum value of 28.9 μA.

**Figure 2:**
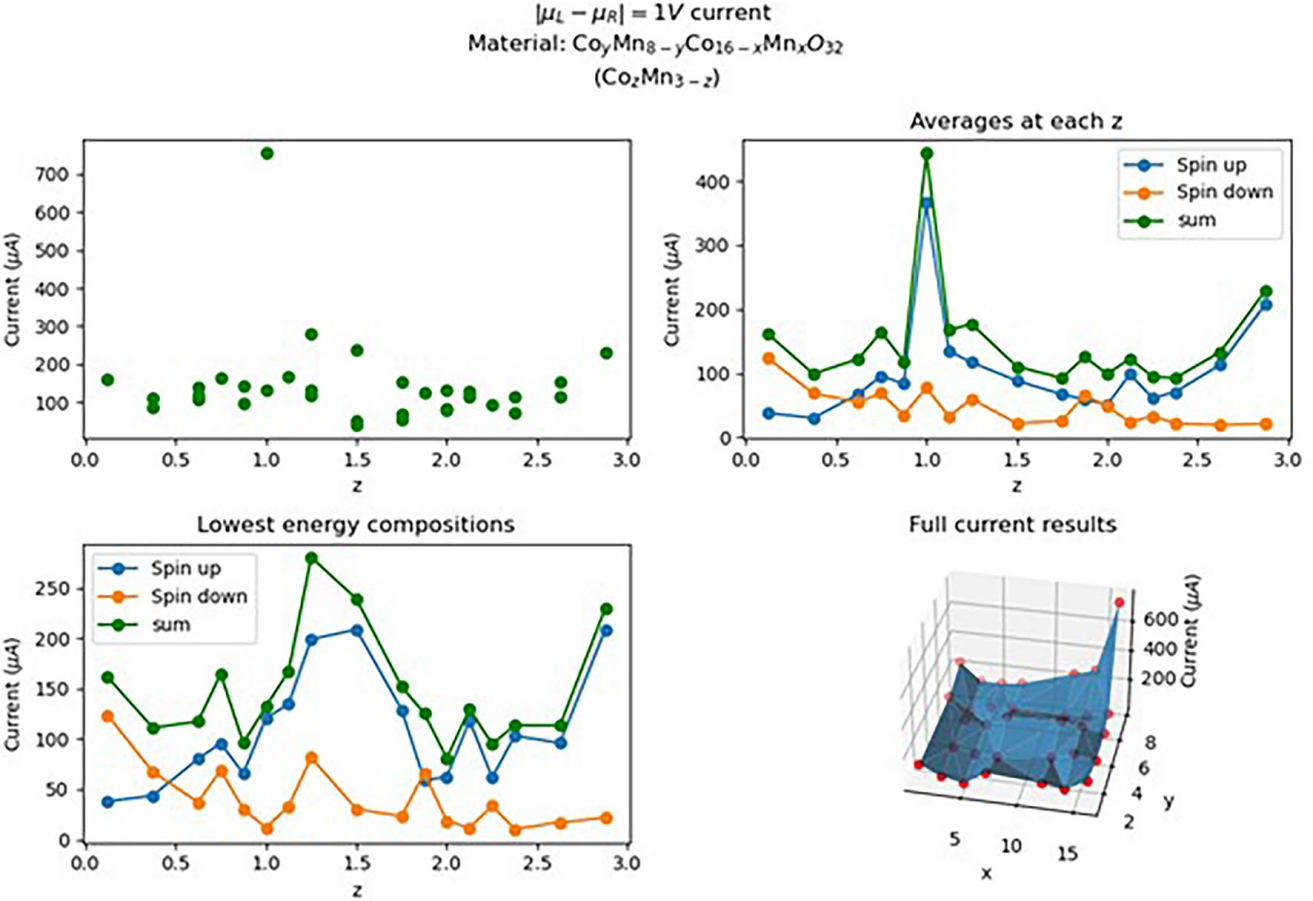
Current calculation results of Co–Mn–O compositions. Top left: all samples results as a function of z. Top right: average results per z. Bottom left: results of currents for the lowest energy sample at each z composition. Bottom right: full results (red dots) for each sample as a function of (x, y) values.

The results for all Co_z_Mn_3−z_O_4_ compositions in the database are illustrated in Fig. 2 In the top left plot, we can see an outlier that produces a current of 755.4 μA, which corresponds to the CoMn_2_O_4_ (Co_8_Mn_0_Co_0_Mn_16_O_32_) composition. Analysis of the band structure of this composition reveals very wide bands for the spin-up part. The MAE of TB fitting for these bands was 0.035eV, the largest in this dataset, as shown by the single bar on the left histogram in Figure S1.

Examining the bottom left plot in Fig. 2, we see the current of the lowest energy structure at each z value. These samples are the most stable compositions and are more likely to be found in the lab. The plot has a triangular shape with a peak at z = 1.25–1.5, which is consistent with the experimental results on Co_z_Mn_3−z_O_4_ nanoparticles from the work of Bhargava et al.^15^ However, these calculations provide a higher limit to the experimental measurements due to additional conditions that may hamper conductivity, such as synthesis process (for example, temperature) and grain boundaries The main difference is that the experiment measurements were done on polycrystalline material and not a single crystal. However, the authors were able to separate the contributions of grain boundaries and intra-particle conductivities and measure the conductivity of a single grain. Therefore, the high concentration of grain boundaries in the experiment is not the reason for different conductivity magnitudes in the simulations and likely the varying synthesis pathways have a contribution.

An examination of the compositions containing nickel cations in the database reveals that a high concentration of Ni induces elevated levels of current. This phenomenon can be observed in Figs. S3–S5. In order to better understand this behavior, we analyzed the band structures, density of states, and transport functions of all datasets containing Ni. It was determined that, in compositions with high concentrations of Ni, the oxygen states are prevalent, as opposed to compositions without nickel where the states in proximity to the Fermi energy are dominated by other cation states. The prevalence of oxygen states is particularly notable in the occupation of p orbitals, which are less localized than the d orbitals of metal oxides and facilitate easier electron transfer between orbitals^16^. Additionally, it was observed that the bands in materials with high concentrations of Ni are wider, which is consistent with high transfer integrals and thus high conductivity (example results in Figs. S6-S8).

This aligns with some experimental data where materials with higher nickel content generally exhibit higher conductivity. It has been demonstrated that nickel doping increases the conductivity of α-Fe_2_O_3_, thus contributing to improved electron transfer ability and reduced charge carrier recombination^17^. There is also evidence that Fe–NiOOH are more conductive than FeOOH^18^. Although these materials have distinct structure from spinel oxide, the effect of Ni addition may be general where a similar behavior may be observed also for spinels. Indeed, a study on NixCo_3−__x_O_4_, indicates a rise in electrical conductivity as the nickel content increases from x = 0 to x = 1, followed by a decline for x > 1 due to the creation of more phases^19^. Furthermore, another work on Nickel doped Cobalt spinel oxide thin films shows decrease in the band gap with increasing nickel content, implying higher electronic conductivity^20^.

### Predicting current from composition

This section presents the performance of the models that were trained to predict the electrical current in spinel oxides based on their compositions, where all models were trained and tested on the dataset that was interduce above. We compare five machine learning algorithms: kernel ridge regression (KRR), support vector regression (SVR), random forests (RF), neural networks (NN), and ensemble of all algorithms. These algorithms are commonly used for supervised learning tasks as we are dealing here. 80% of data is training, 20% is testing. Five-fold cross-validation was used to determine the hyperparameters of the models. Performance is evaluated using MAE and R^2^ on the test set.

The input vectors for these models were defined as a vector of size eight, with the first four cells indicating the specie and composition of A atoms and the last four cells indicating the specie and composition of B atoms. The value in each of the first four cells represents the number of A atoms divided by eight (as there are eight tetrahedral sites), with each cell representing a different species in this order: Mn, Fe, Co, and Ni. This means that only one of the first four cells will have a non-zero value, indicating the specie of A atoms and their relative amount in the tetrahedral sites. The last four cells follow the same logic, but for B atoms, with their value being the number of B atoms divided by 16 (as there are 16 octahedral sites).

**Figure 3:**
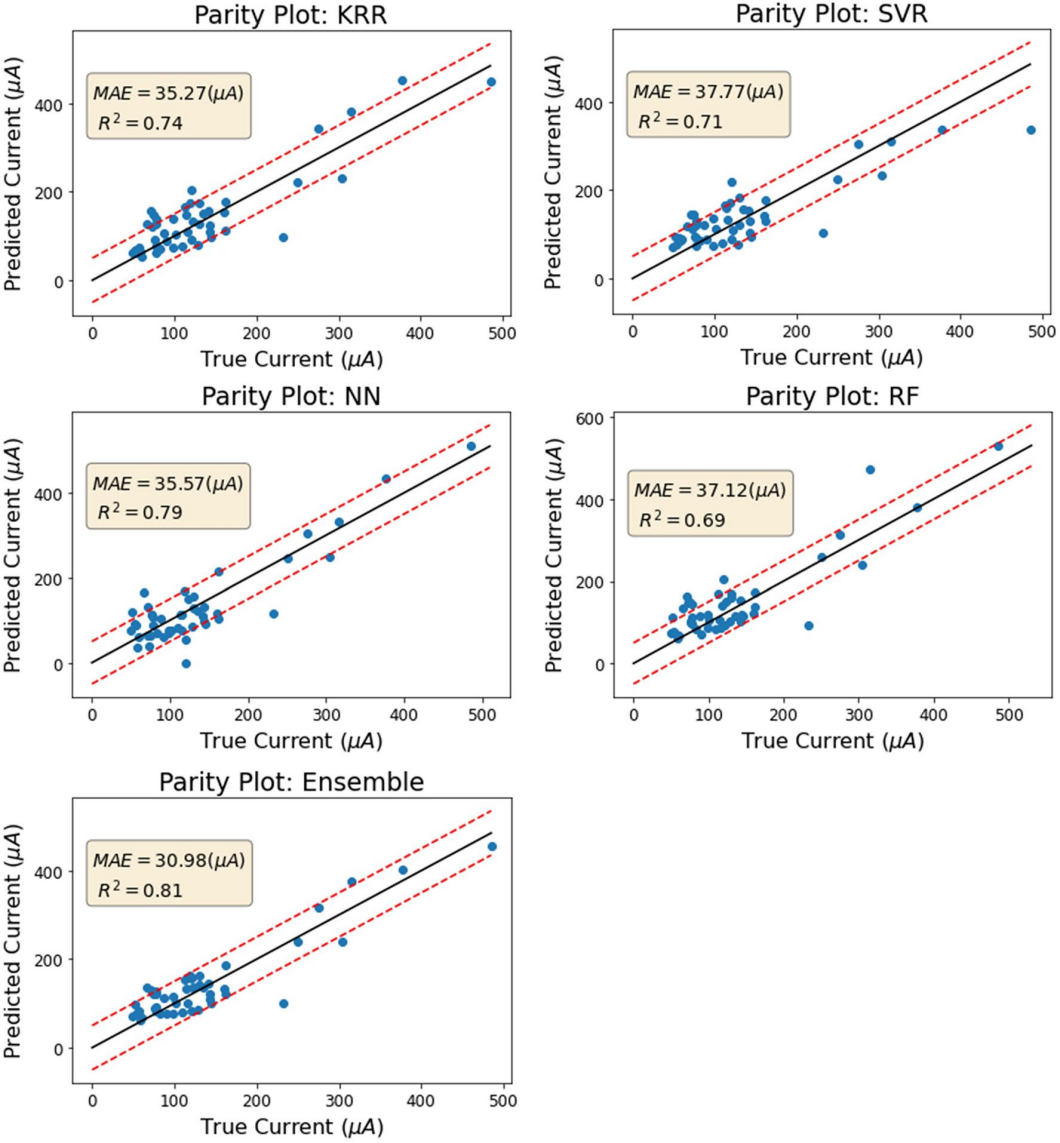
The performances of all five trained machine learning models on the test set.

**Figure 4:**
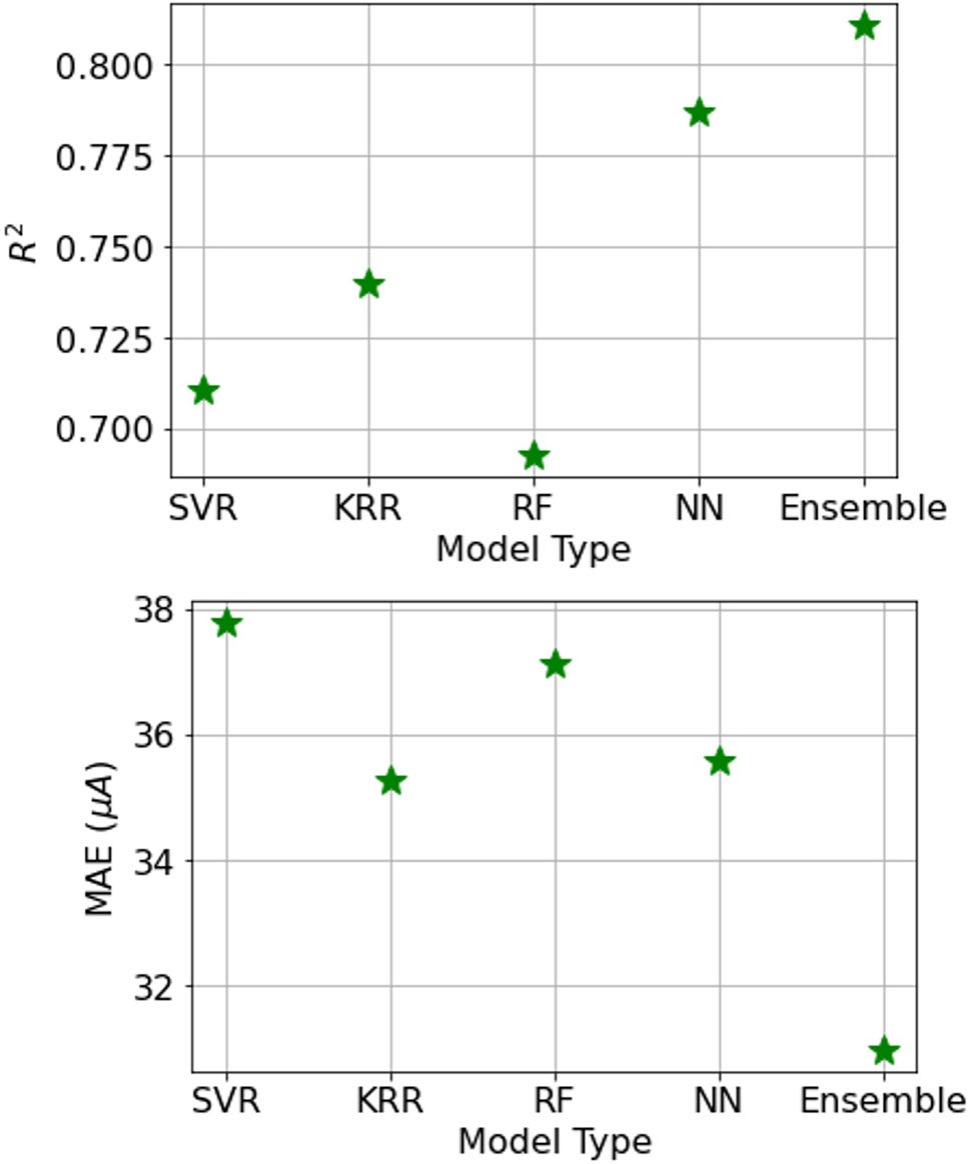
Top: The R2 results of all five trained machine learning models. Bottom: The MAE results of all five trained machine learning models.

All four initial algorithms (KRR, SVR, RF, and NN) predicted the current of the test set samples with a mean absolute error of 35–37 μA and R-squared values between 0.69 and 0.79 (as shown in Fig. 3 and Fig. 4). Generally, all four models performed well and with similar accuracy, with the ensemble performing the best. The ensemble model, which takes the average output result of the other four algorithms, improves the mean absolute error to 30.98 μA and R-squared to 0.81 (as shown in Fig. 4). This can be explained by the fact that some of the models predict certain samples more accurately than others, and taking the average of the results compensates for errors in individual models. As a benchmark to evaluate the performance of our ML predictors, we used the scenario where the models predict the mean value of the dataset for all compositions, resulting in a predicted current of 149.7 μA for every input. In this case, the mean absolute error is 72.56 μA. As we will see in the next section, the predictions of the ML models are significantly more accurate than this baseline. Overall, A small dataset (190 samples) does not necessarily hinder the creation of an accurate predictive model, as long as the training set covers a majority of the relevant domain.

The high accuracy of the model is noteworthy, however, there is room for improvement. The similarity in accuracy between different models suggests that they are well-suited to the data and additional fine-tuning of the algorithms’ hyperparameters will not result in significant changes. The source of error may be attributed to inconsistencies in the target values (currents) in the dataset, which the models are unable to capture. As the NEGF and Landauer methods were applied consistently across all samples, it is unlikely that this is the cause. Instead, the inconsistencies may originate from the DFT calculations used to produce the band structures for current calculations. The DFT + U calculation scheme used in this study selected U values for transition metals based on previous works, however, there are multiple U values for each transition metal found in literature and these values are optimized for specific environments that can affect the value of U. As a result of choosing consistent U values for each transition metal across all compositions in the dataset, it is possible that the U value causes inconsistencies in the resultant band structures of some compositions. This assumption can be verified by performing band structure calculations using a hybrid functional that does not rely on empirical parameters, but due to the time-consuming nature of this task and the already high accuracy of the ML model, such calculations were not performed in this study.

### Bandwidth model

**Figure 5:**
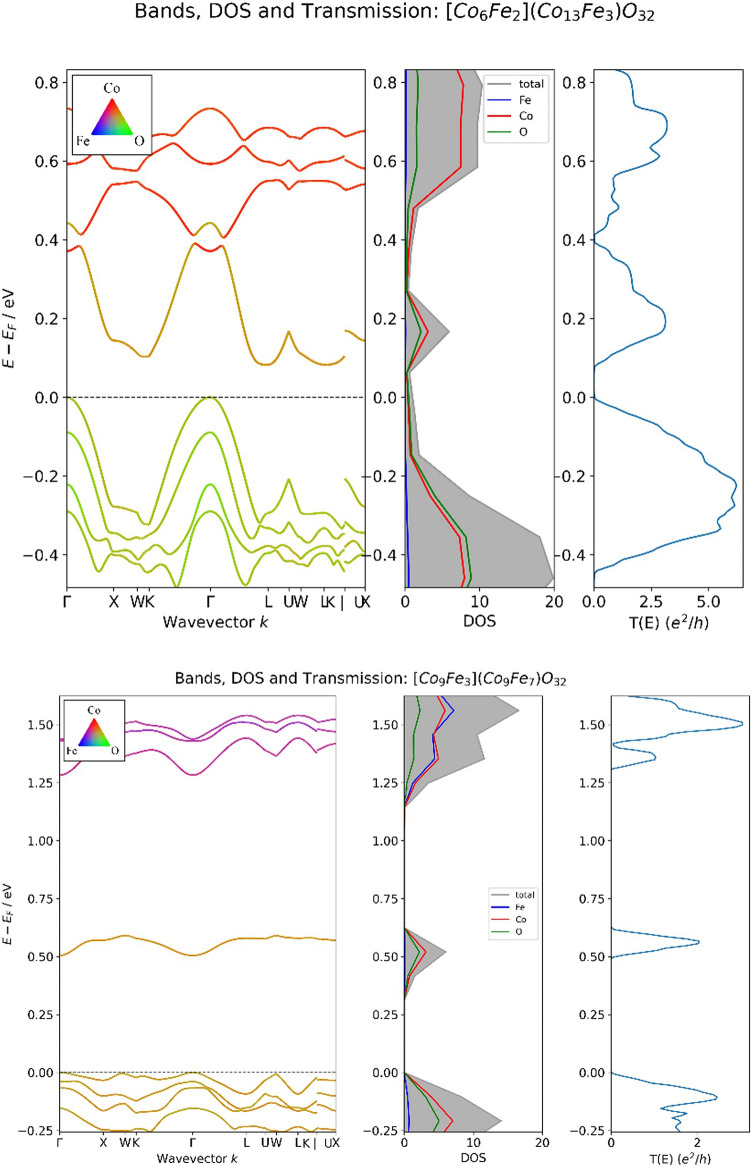
Top: band structure, DOS, and transmission function of wide bandwidth sample. The current that passes through this sample under 1 V bias is 208.79μA. Bottom: band structure, DOS, and transmission function of narrow bandwidth sample. The current that passes through this sample under 1 V bias is 44.9 μA.

Upon examination of the band structures, it is evident that samples with wider bands produce higher currents after the NEGF calculation (as shown in Fig. 5 top). Conversely, samples with narrower bands result in lower currents (as shown in Fig. 5 bottom). This is because wider bands imply a higher coupling between states, allowing electrons to flow with less resistance. Coupling between states is related to the transfer integral in tight-binding Hamiltonians, and as the transfer integral term is larger, the bandwidth is wider^21–23^. To leverage this physical property, we trained a neural network and XGboost (XG) algorithms that use the widths and minimum energy of the eight bands in the band structure to predict the current under 1 V bias for a given sample.

The dataset for this model consists of two band structures (one for each spin) from each of the 190 compositions that were calculated, resulting in a total of 380 samples of band structures. 20% of the dataset was held out for evaluating the model, while the remaining 80% was used for training the two models. The input vector was defined as [bw_1_, Emin_1_, bw_2_, Emin_2_, …, bw_8_, Emin_8_], where bw_i_ is the bandwidth of band i and Emin_i_ is the minimum energy of band i. The bandwidth is the difference between the maximum energy and minimum energy in the band. The output to be predicted is the calculated current under 1 V bias. The hyperparameters for the XGboost and neural network models are listed in the SI.

**Figure 6:**
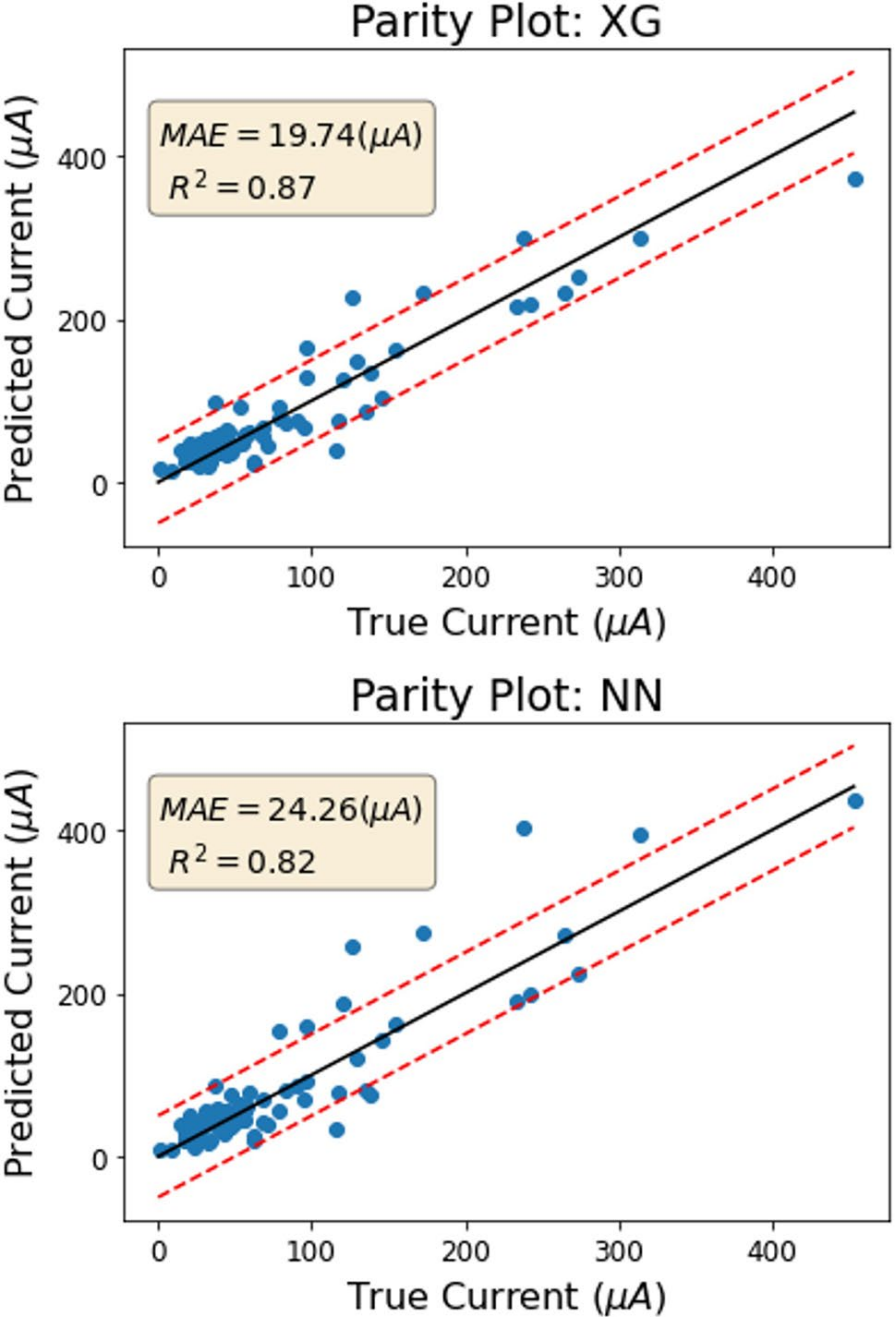
Top: test set results of the XGboost model to predict current based on bandwidths for spinel oxides. Bottom: test set results of the neural network model to predict current based on bandwidths for spinel oxides.

The XGboost algorithm predicted the current generated by each electronic structure with an MAE of 19.74 µA, which is 56% more accurate than the results of the ensemble model in the previous predictor (as shown in Fig. 6 top). The R-squared value of 0.87 indicates a strong linear correlation between the predicted and actual values of the current. The performance of the neural network was slightly lower (as shown Fig. 6 bottom), but both models performed well overall. This model demonstrates the ability to predict the physical property of conductivity based on electronic structure inputs such as bandwidths. As a result, it would be possible to use this trained model to analyze large sets of band structures and determine their conductivities, thus saving time by avoiding the full Green's function procedure.

### Band gap predictor

**Figure 7:**
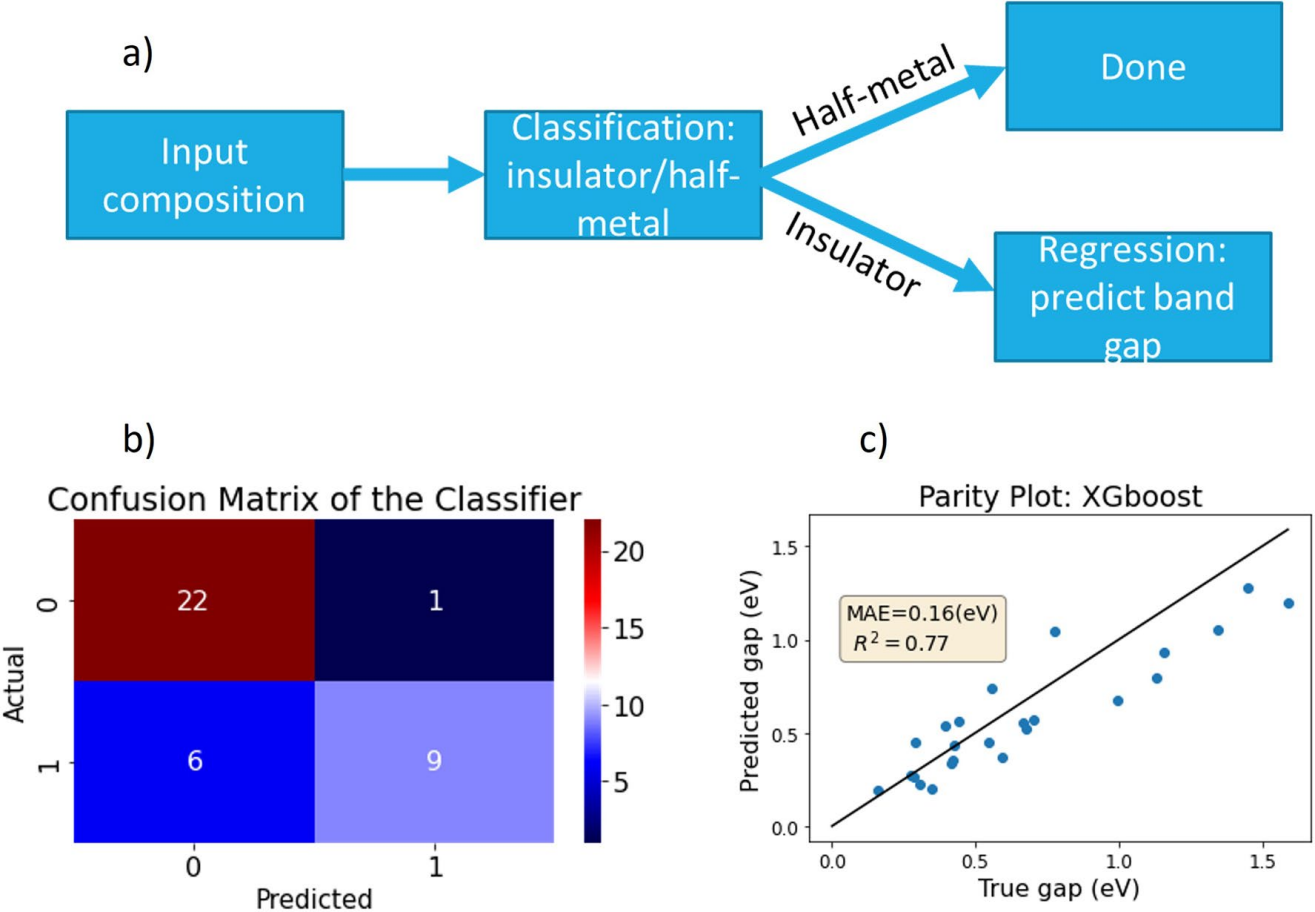
(**a**) Band gap predictor scheme. (**b**) Confusion matrix of the test results for the semiconductor/half-metal classifier. (**c**) Parity plot of the band gap regression model.

Predicting the band gap of materials has attracted significant interest due to the potential of selecting the appropriate materials for semiconductor device engineering^24^. In this part, we train a two-step model; first, it classifies whether the composition is a half-metal or a semiconductor (as shown in Fig. 7a). Then, if it is predicted to be a semiconductor, a regressor predicts the value of the band gap. If it predicted to be a metal, the process is done. The classifier is an XGboost algorithm that was trained and cross-validated on the training set and then tested on the test set. The regression model is also based on XGboost and was trained on a set of 93 out of the 117 semiconductors in the dataset, with the other 24 samples being used for testing. The optimized hyperparameters of the two models are summarized in SI.

The input vector for these models was defined as [A, B, y, x], representing the composition of each sample. Where A and B are the atomic numbers of the A and B atoms in the sample, and x and y are the stoichiometric numbers according to the spinel formula. The inputs were standardized by subtracting the mean value and scaling to unit variance. This is a common pre-processing step for many machine learning algorithms, as it helps in training the models and prevents one feature in the input vector from dominating others (i.e. atomic numbers are larger than the x, y values).

The performance of the classifier can be viewed in Fig. 7a and Table S1, where '0' represents a semiconductor and ‘1’ represents a half-metal material. The key metric here is the accuracy, which is 82% (correct predictions out of the total samples), indicating very good results for such a small dataset. The performance of the regressor is presented in Fig. 7c, where a good linear fit is observed between the true values and predicted values of the band gap, as well as a low mean absolute error (MAE). Therefore, this model can provide a reliable estimate of the band gap for spinel oxides in a matter of seconds, without the need for full DFT calculations.

## Conclusions

The aim of this research was to investigate the conductivity properties of spinel oxide materials used in electrochemical applications. Models were developed aiming to predict electronic conductivity based only on their compositions, as electronic properties can be adjusted by changing stoichiometry and chemical composition. To achieve this, three methods were used: (1) DFT calculations to obtain the geometric and electronic structure of 190 different spinel oxides, (2) Quantum transport calculations including fitting of a tight-binding Hamiltonian to band structures, and (3) ML algorithms to predict conductivity and band gap.

According to the results spinel oxide compounds with Ni cations showed higher current values due to the domination of delocalized oxygen p states over transition metal d states. This behavior suggests the potential for more effective charge transport by adding nickel atoms. In terms of practical applications, these high conductivity materials could be highly beneficial for energy conversion devices. High conductivity implies lower resistive losses, which can improve the efficiency of energy conversion and transmission processes. Specifically, in devices such as fuel cells and batteries, materials with high electronic conductivity can enhance the device performance by facilitating faster electron transport, leading to higher power densities. However, it’s important to note that while our models predict high conductivity for these materials, further experimental validation would be necessary to confirm these predictions and to assess their feasibility for use in practical applications. Additionally, machine learning models were able to predict band gap and conductivity based solely on spinel composition with a relatively small database. A model leveraging bandwidth as a fingerprint was developed to predict current and can save time in NEGF calculations. Moreover, we believe that the findings concerning the utilization of bandwidths as a distinctive material fingerprint have paved the way for the creation of robust predictive models, enhancing material research.

## Methods

In the following section, we will give details about the methodologies that make up our proposed workflow for predicting the conductivity and band gap of spinel oxide materials: DFT for band structure calculation, principal layer, Green’s functions, Landauer formalism for electric conductivity, and machine learning and band structure fitting to tight binding Hamiltonian.

### DFT for band structure calculation

**Figure 8:**
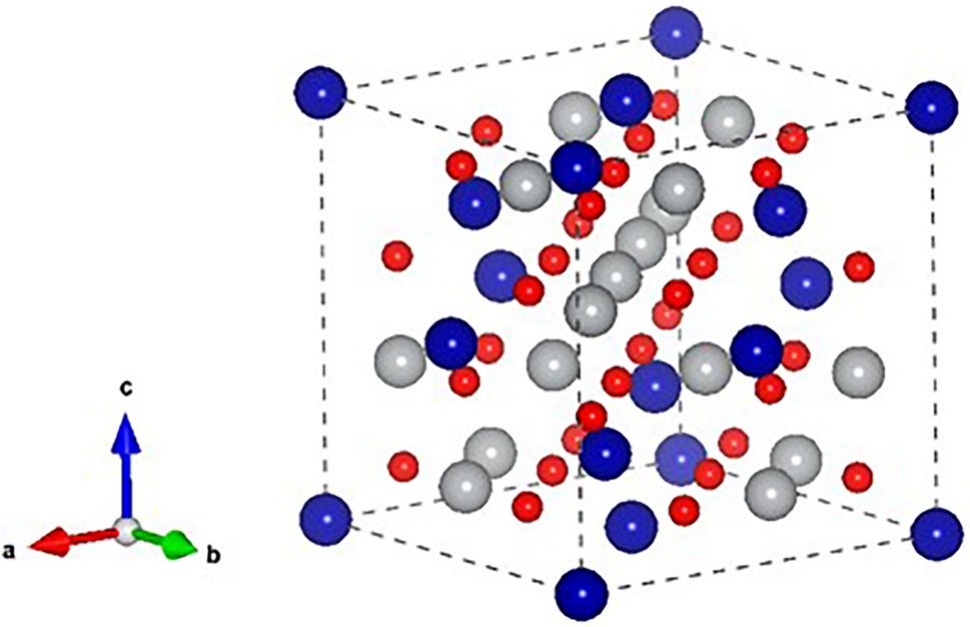
An example of normal spinel oxide unit cell geometry (Co_16_Ni_8_O_32_). The red spheres are oxygen ions, gray are nickel ions in tetrahedral sites and blue are cobalt ions in octahedral sites.

All DFT calculations were done using the Vienna ab-initio simulation package (VASP)^25^ with the Perdew–Burke–Ernzerhof (PBE) functional^26^. We align the spins in a ferrimagnetic arrangement with Néel collinear configuration, where the magnetic moment of tetrahedral cations is opposing that of octahedral cations. Following convergence tests, the calculations for the cubic cells utilized a 600 eV plane wave energy cut-off and a 3 × 3 × 3 k-point mesh, centered at the Г-point. The self-consistent electronic minimization employed a 0.1meV threshold and ionic relaxation had a force threshold of 0.01 eV/Å. The band structure was calculated using k-points along high symmetry lines, selected based on the implementation in the pymatgen^27^ library as described in ref^28^. The k-point file is available in the SI. The Hubbard U values applied to Mn, Co, Fe, Ni, and O atoms were 3.9, 3.5, 4.5, 5.5, and 0 eV, respectively, where all U values were used based on previous literature works^29–35^. The unit cell of all the spinels in the dataset consists of 56 atoms, 32 of which are oxygen, 16 of which are transition metal ions in tetrahedral sites and 8 transition metal ions in octahedral sites (Fig. 8). The dataset contains 190 different combinations of this cell as explained later in the results.

### Principal layer, Green’s functions, and Landauer formalism for electric conductivity

For calculating the current, we followed the well-established approach of Landauer–Büttiker for quantum transport^36,37^. In this formalism, we postulate that there is a potential between two electron reservoirs that possess an equilibrium distribution and has dissimilar chemical potentials. These reservoirs act as sources and drains of electrons for the left and right electrodes, respectively. The device, which we intend to evaluate its current, constitutes the scattering region located between the electrodes. The variance in the electrochemical potentials of the reservoirs represents the applied bias on the scattering region. The Landauer–Büttiker is

1 $$I=\frac{2e}{h}\underset{-\infty }{\overset{\infty }{\int }}T\left(E\right)\left[f\left(E-{\upmu }_{L}\right)-f\left(E-{\upmu }_{R}\right)\right]dE$$

where $f\left(E-{\upmu }_{L}\right)$ and $f\left(E-{\upmu }_{R}\right)$ are the Fermi–Dirac electron distributions with ${\upmu }_{L}$ and ${\upmu }_{R}$ the chemical potentials of the left and right electrodes, respectively. $T\left(E\right)$ is the transmission function of an electron from left to right electrodes through the scattering region. In this setup the system is divided into principal layers that interact only with the nearest neighbors’ layer and each layer is described by a tight-binding Hamiltonian, ${H}_{i}$, where i is the layer number. Since each electrode is semi-infinite, this approach introduces an infinite problem. Fortunately, this problem is solved by using Green’s function. The scattering region which must interest us is a finite region that can be obtained by

2 $${{\varvec{G}}}_{{\varvec{S}}}={\left(\left(E+i\eta \right){{\varvec{S}}}_{{\varvec{S}}}-{{\varvec{H}}}_{S}-{\boldsymbol{\Sigma }}_{L}-{\boldsymbol{\Sigma }}_{R}\right)}^{-1}$$

where E is the energy of the system and $\eta$ is a small number. ${{\varvec{S}}}_{{\varvec{S}}}$ is the overlap matrix of the scattering region The overlap matrix terms are defined as the overlap integral between associate vectors of the Hamiltonian basis. ${{\varvec{H}}}_{{\varvec{S}}}$ is a block matrix representing the scattering region, and the effect of the semi-infinite electrodes is contained through the finite self-energy matrices ${{\varvec{\Sigma}}}_{{\varvec{L}}\backslash {\varvec{R}}}$ of electrode left (L) and right (R) electrodes.

The transmission formula can be obtained from the Landauer–Büttiker formalism and quantum scattering theory^36–39^, and it is given by

3 $${\mathbf{T}}\left( {\mathbf{E}} \right) = {\text{Tr}}\left\{ {{{\varvec{\Gamma}}}_{{\mathbf{L}}} {\mathbf{G}}_{{\mathbf{S}}}^{\dag } {{\varvec{\Gamma}}}_{{\mathbf{R}}} {\mathbf{G}}_{{\mathbf{S}}} } \right\}$$

where ${\Gamma }_{L\backslash R}$ is called the broadening matrix and it is given by the expression

4 $${{\varvec{\Gamma}}}_{{{\mathbf{L}}\backslash {\mathbf{R}}}} = {\text{i}}\left( {{{\varvec{\Sigma}}}_{{{\varvec{L}}\backslash {\varvec{R}}}} -{\varvec{\varSigma}}_{{{\varvec{L}}\backslash {\varvec{R}}}}^{\dag } } \right)$$

A technique for deriving finite self-energy matrices is available in the literature^40,41^. This procedure results in the following terms for the left electrode’s self-energy.

5 $$\hat{\user2{\Sigma }}_{{\varvec{L}}} = {\varvec{H}}_{{\user2{L^{\prime}},{\varvec{L}}}}^{\dag } {\varvec{g}}_{{\varvec{L}}} {\varvec{H}}_{{\user2{L^{\prime}},{\varvec{L}}}}^{{}}$$

where ${{\varvec{H}}}_{{{\varvec{L}}}^{\boldsymbol{^{\prime}}},{\varvec{L}}}$ is the coupling Hamiltonian between the scattering region and the left electrode. And ${{\varvec{g}}}_{{\varvec{L}}}$ is given by a recursive formula,

6 $$g_{L}^{{\left( {n + 1} \right)}} = \left[ {{\varvec{H}}_{{\varvec{L}}} - {\varvec{H}}_{{\user2{L^{\prime}},{\varvec{L}}}}^{\dag } {\varvec{g}}_{{\varvec{L}}}^{{\left( {\varvec{n}} \right)}} {\varvec{H}}_{{\user2{L^{\prime}},{\varvec{L}}}}^{{}} } \right]^{ - 1}$$

The initial guess is ${{\varvec{g}}}_{{\varvec{L}}}^{\left({\varvec{n}}\right)}={{\varvec{H}}}_{{{\varvec{L}}}^{\boldsymbol{^{\prime}}},{\varvec{L}}}$ and the iterative procedure continues until ${\left({{\varvec{g}}}_{{\varvec{L}}}^{\left({\varvec{n}}-1\right)}-{{\varvec{g}}}_{{\varvec{L}}}^{\left({\varvec{n}}\right)}\right)}_{ij}^{2}\le {\updelta }^{2}$, where $\updelta$ is a small number in the order of 1 × 10^−15^, this convergence criterion is achieved within few iterations. A similar procedure is done for the right self-energy (for symmetric electrodes and bias ${{\varvec{g}}}_{{\varvec{L}}}={{\varvec{g}}}_{{\varvec{R}}})$. ${\boldsymbol{\Sigma }}_{{\varvec{L}}}$ takes the size of the scattering Hamiltonian and has a block element only in the top left of the left self-energy matrix and in the bottom right of the right self-energy,

7 $${\boldsymbol{\Sigma }}_{{\varvec{L}}}=\left(\begin{array}{ccccc}{\widehat{\Sigma }}_{L}& 0& \cdots & 0& 0\\ 0& 0& 0& \cdots & 0\\ \vdots & 0& 0& \cdots & \vdots \\ 0& \vdots & \cdots & \ddots & 0\\ 0& 0& \cdots & 0& 0\end{array}\right)$$

**Figure 9:**
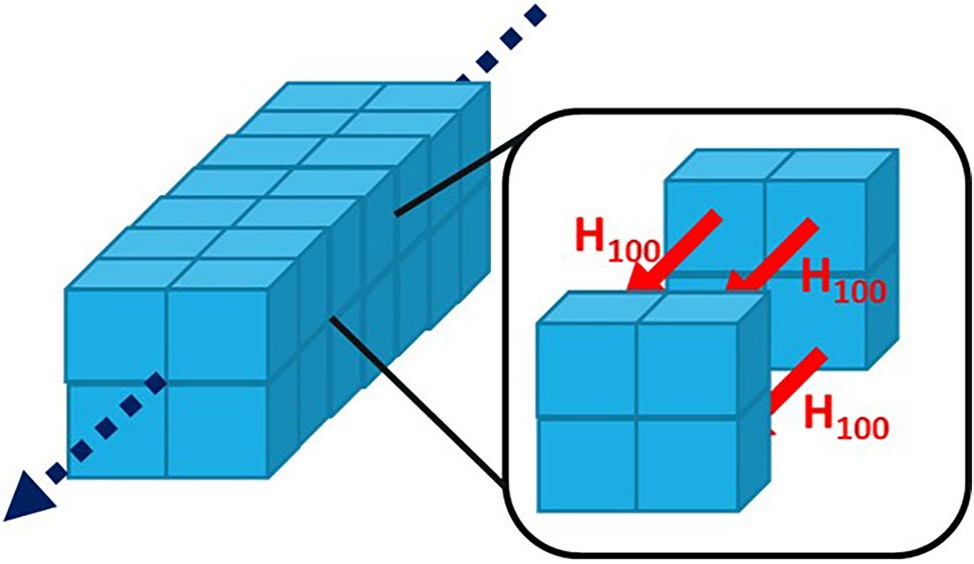
A schematic representation of a wire built from principal layers of four unit cells each. The layers are coupled in the 100 direction.

Our system is divided into the so-called principal layers^42–44^ where each layer consists of four unit cells of spinel oxide as illustrated in Fig. 9. Using only four unit cells is sufficient to capture all the necessary information from the TB Hamiltonians that were adjusted to fit the DFT band structure. The layers are interconnected by a coupling Hamiltonian, effectively forming an endless wire. The Hamiltonian of a single layer of our system was defined as follows

8 $$H_{layer} = \left( {\begin{array}{*{20}c} {{\varvec{H}}_{00} } & {{\varvec{H}}_{010} } & {{\varvec{H}}_{001} } & 0 \\ {{\varvec{H}}_{010}^{\dag } } & {{\varvec{H}}_{00} } & 0 & {{\varvec{H}}_{001} } \\ {{\varvec{H}}_{001}^{\dag } } & 0 & {{\varvec{H}}_{00} } & {{\varvec{H}}_{010} } \\ 0 & {{\varvec{H}}_{001}^{\dag } } & {{\varvec{H}}_{010}^{\dag } } & {{\varvec{H}}_{00} } \\ \end{array} } \right)$$

where the elements in the Hamiltonian matrix are blocks of matrices. The blocks along the diagonal correspond to a tight-binding Hamiltonian (the tight-binding Hamiltonians defined in the next section) of each of the four unit cells within a single layer of spinel oxide. The block matrices along the off-diagonal represent the tight-binding Hamiltonian matrix elements that connect each unit cell with its neighboring cells within the same layer. The subscripts used for H indicate the spatial directions between a unit cell and its adjacent neighbors. The block matrix below defines the coupling between each layer and its neighboring layers,

9 $${H}_{layerCoupling}=\left(\begin{array}{cccc}{{\varvec{H}}}_{100}& 0& 0& 0\\ 0& {{\varvec{H}}}_{100}& 0& 0\\ 0& 0& {{\varvec{H}}}_{100}& 0\\ 0& 0& 0& {{\varvec{H}}}_{100}\end{array}\right)$$

To maintain a manageable size for the Hamiltonians and take advantage of the electrodes being made of the same material, we opted to use only two layers to represent our material in the scattering region. This approach essentially yields an infinite material through which the current can flow. The Hamiltonian for the scattering region is expressed as follows:

10 $$H_{s} = \left( {\begin{array}{*{20}c} {{\varvec{H}}_{{{\varvec{layer}}}} } & {{\varvec{H}}_{{{\varvec{layerCoupling}}}} } \\ {{\varvec{H}}_{{{\varvec{layerCoupling}}}}^{\dag } } & {{\varvec{H}}_{{{\varvec{layer}}}} } \\ \end{array} } \right)$$

The size of the scattering Hamiltonian is composed of 2 × 2 blocks representing two layers and their connection, and each block is composed of 4 × 4 block matrices that include ${{\varvec{H}}}_{00},{{\varvec{H}}}_{100},{{\varvec{H}}}_{010},{{\varvec{H}}}_{001}$ , where these matrices have the size of 8 × 8 (since in this work we fitted to eight bands). Thus, in total the size of ${H}_{s}$ is 64 × 64.

### Machine learning

The project emphasizes material property prediction and follows a workflow suited for such a task. The workflow includes the following steps: building a dataset by collecting and cleaning data and dividing it into a train and test set, selecting features, training the model by selecting an appropriate model, tuning hyperparameters, and optimizing it. The model's performance is evaluated on the test set and compared to other models, and finally, the model is used for making predictions.

To prepare for model training and testing, the dataset is first divided into two sets: a training set and a test set. The latter is used to evaluate the model's ability to predict properties on new data that it has not seen during training. It's a mistake to train and test on the same data since the model can overfit the training data and perform poorly on new data. A standard approach is to partition 80% of the dataset for training and reserve the remaining 20% for testing.

To set up a dataset for machine learning (ML) models, it should be divided into features and target variables. Features are independent variables grouped into a vector that serves as input for the ML model. Choosing informative and independent features is crucial for model performance. A single input vector represents a sample, and the order of features must remain consistent. The target variables are the properties to be predicted. For the materials in this project, A and B elements and stoichiometric numbers x and y from the formula A_y_B_y−8_A_16−x_B_x_O_32_ are the most representative features. Oxygen has a constant value and doesn't add information to the feature vector. The A and B atoms are represented by their atomic number values: Mn = 25, Fe = 26, Co = 27, and Ni = 28. Features normalization and scaling are important to ensure a proper functioning of some ML algorithms and to speed up gradient descent. In this project, we applied standardization using Scikit-learn module^45^, which involves removing the mean value and scaling to unit variance.

For the regression problems, which are predicting current from compositions, predicting current from bandwidth and prediction of bandgaps, we have chosen to utilize five different machine learning algorithms for comparison of their performance. For predicting current from composition these algorithms include kernel ridge regression (KRR), support vector regression (SVR), random forests (RF), neural networks (NN), and an ensemble model which takes the average result of all other four algorithms. For the prediction of current from bandwidth and for the bandgap prediction we used the XGboost algorithm^46^. These algorithms were chosen as they each have unique strengths in handling different types of data and relationships. Specifically, NN were chosen due to their ability to model complex, non-linear relationships, which is often the case in predicting material properties like electronic conductivity and band gaps; SVR was selected for its effectiveness in high-dimensional spaces, which is common in material science data. It also has strong theoretical foundations in statistical learning theory; KRR was chosen for its ability to handle non-linear relationships through the use of kernel functions. It also has the advantage of being less sensitive to outliers; RF was selected for its robustness and ease of use. It performs well with both linear and non-linear data.

ML models are functions defined by parameters. During training, the model optimizes the parameters to fit the data. Hyperparameters, like regularization values or architecture of a neural network, are tuned by the user. To avoid overfitting and ensure models perform well with new predictions, cross-validation procedures are used. The training set is split into k-folds, and the model is trained on k-1 folds and evaluated on the remaining fold (validation set) k times. The average performance is reported, and once hyperparameters are tuned, the model is trained on the whole training set and tested. In evaluating the model, selecting an appropriate metric is crucial. For this work, we have chosen the mean absolute error (MAE) as it provides a natural measurement of the accuracy of a model that predicts physical values such as current. Additionally, we have used R^2^ to measure the linearity between the predicted values of the models and the true values that were calculated.

### Band structure fitting to tight binding Hamiltonian

Traditional tight-binding fitting schemes entail tuning numerous parameters that represent the atomic structure and do not always yield precise results^47^. A more common approach is utilizing “Wannier functions”, obtained by transforming extended Bloch functions from DFT calculations, which derive tight-binding parameters from ab-inito results without the need for fitting^48,49^. However, this method demands extensive system knowledge and a trial-and-error process due to the numerous parameters involved. Our objective is to rapidly and precisely compute the tight-binding Hamiltonian for approximately two hundred material samples.

Our approach follows the method proposed by Wang et al., where a parametrized tight-binding Hamiltonian is obtained by employing the back-propagation technique to fit the real-space tight-binding matrices to the DFT band structure^50^. Here we implemented the same approach but with pyTorch^51^ library instead of TensorFlow^52^ as done by the original authors. In TB theory the reciprocal space Hamiltonian for a desired K vector is,

11 $${H}^{TB}(k)={\sum }_{R}{e}^{ik\cdot R}{H}^{R}$$

where R is a lattice vector of selected real-space Hamiltonian matrices and ${H}^{R}$ is the corresponding TB Hamiltonian. H^0^ (R = (000)) is the real-space TB Hamiltonian matrix of the unit cell, ${H}^{R}\ne 0$ (R = (100), (010), (001), etc.) are the Hamiltonian matrices that couple the unit cell Hamiltonian to the adjacent unit cells in the direction of R vectors. The TB band structure is obtained from the eigenvalues ${{\varepsilon }^{TB}}_{n,k}$ for every desired k vector via,

12 $${H}_{k}^{TB}{\psi }_{n,k}^{TB}={{\varepsilon }^{TB}}_{n,k}{\psi }_{n,k}^{TB}$$

where ${{\varepsilon }^{TB}}_{n,k}$ is the energy of the n-th band at reciprocal vector k. To fit the TB bands to the DFT bands, the mean squared error loss L between the TB and DFT eigenvalues is minimized,

13 $$L=\frac{1}{N}\frac{1}{{N}_{k}}{{\sum }_{i=1}^{N}{\sum }_{k}{\left({\varepsilon }_{i,k}^{TB}-{\varepsilon }_{i,k}^{DFT}\right)}^{2}}$$

The loss is computed for all bands and k-points, where N represents the total number of bands and N_k_ represents the number of sampled k-points. The TB Hamiltonian’s parameters (H^R^) are updated iteratively using the Adam gradient descent algorithm^53^. The back-propagation procedure is used to calculate the derivative of the loss with respect to H^R^s. The gradient descent algorithm seeks to minimize the loss by moving in the direction of the steepest descent, which is opposite to the direction of the gradient, according to the general formula,

14 $${H}_{l+1}^{R}={H}_{l}^{R}-\alpha \frac{\partial L}{\partial {H}_{l}^{R}}$$

The variable "l" represents either an epoch number (epoch defines the number of times the entire data set, in this case all the k points, has worked through the learning algorithm) or a single iteration over all k points. In this context, an epoch is considered complete after the loss function has accumulated all the eigenvalues in the band structure, which occurs after calculating all ${H}^{TB}(k)$ values. The derivative of the loss with respect to H^R^ is given with matrix derivative algorithmic differentiation rules^54^.

The fitting process has a predefined numerical threshold (means squared error < $3\cdot {10}^{-5} {\text{eV}}^{2}$, as defined in Eq. 13) for the loss function, which serves as a criterion to terminate the fitting. Additionally, we introduced another criterion that halts the fitting if the loss increases for more than ten epochs due to spikes or jumps in the loss function during training. If the loss was already low, we consider the results of the fitting process. However, if the loss was high, we re-run the process with slightly different initial random weights in the Hamiltonians.

To determine the R vectors for the fitting process, we conducted tests with various vectors, up to 24 in total. After experimentation, we concluded that the three main directions and their opposite directions were sufficient for achieving a highly accurate fitting of the bands. The size of the matrices used in the fitting process is defined by the number of bands being fitted. For instance, for eight bands, the matrix size is 8 × 8.

## Supplementary Information

Supplementary Information 1.

Supplementary Information 2.

## Data Availability

The data generated or analyzed during this study are included in this published article (and its Supplementary Information files).
