# Supplementary material for: From density functional theory to machine learning predictive models for electrical properties of spinel oxides
Source: Sci Rep. 2024 May 27;14:12150. doi: 10.1038/s41598-024-62788-4 (PMC11637019; doi:10.1038/s41598-024-62788-4)
Supplement: Supplementary file 2 — Supplementary Information 2. [file 41598_2024_62788_MOESM2_ESM.docx]

**From Density Functional Theory to machine learning predictive models for electrical properties of spinel oxides**

*Yuval Elbaz, Maytal Caspary Toroker**

*Department of Materials Science and Engineering, Technion – Israel Institute of Technology, Haifa 3600003, Israel*

*maytalc@technion.ac.il

**Supporting Information**


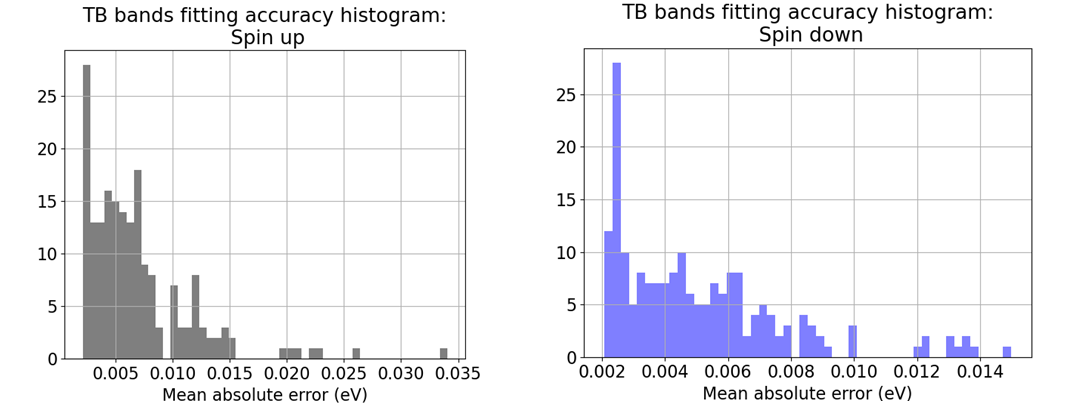


Figure S1.MAE of the fitting accuracy to TB Hamiltonian of spin up (left) and down (right) bands of all compositions in the dataset..


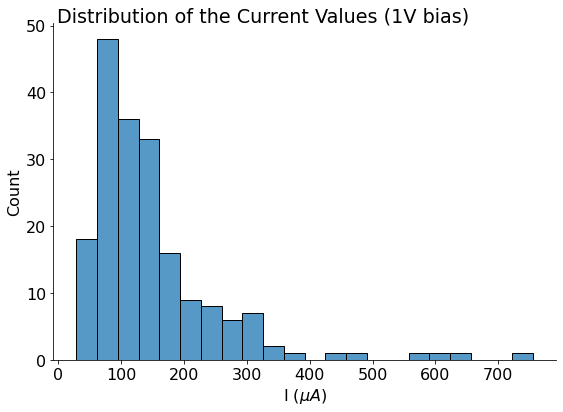


Figure S2. Distribution of the current values in the dataset at a bias of 1V.


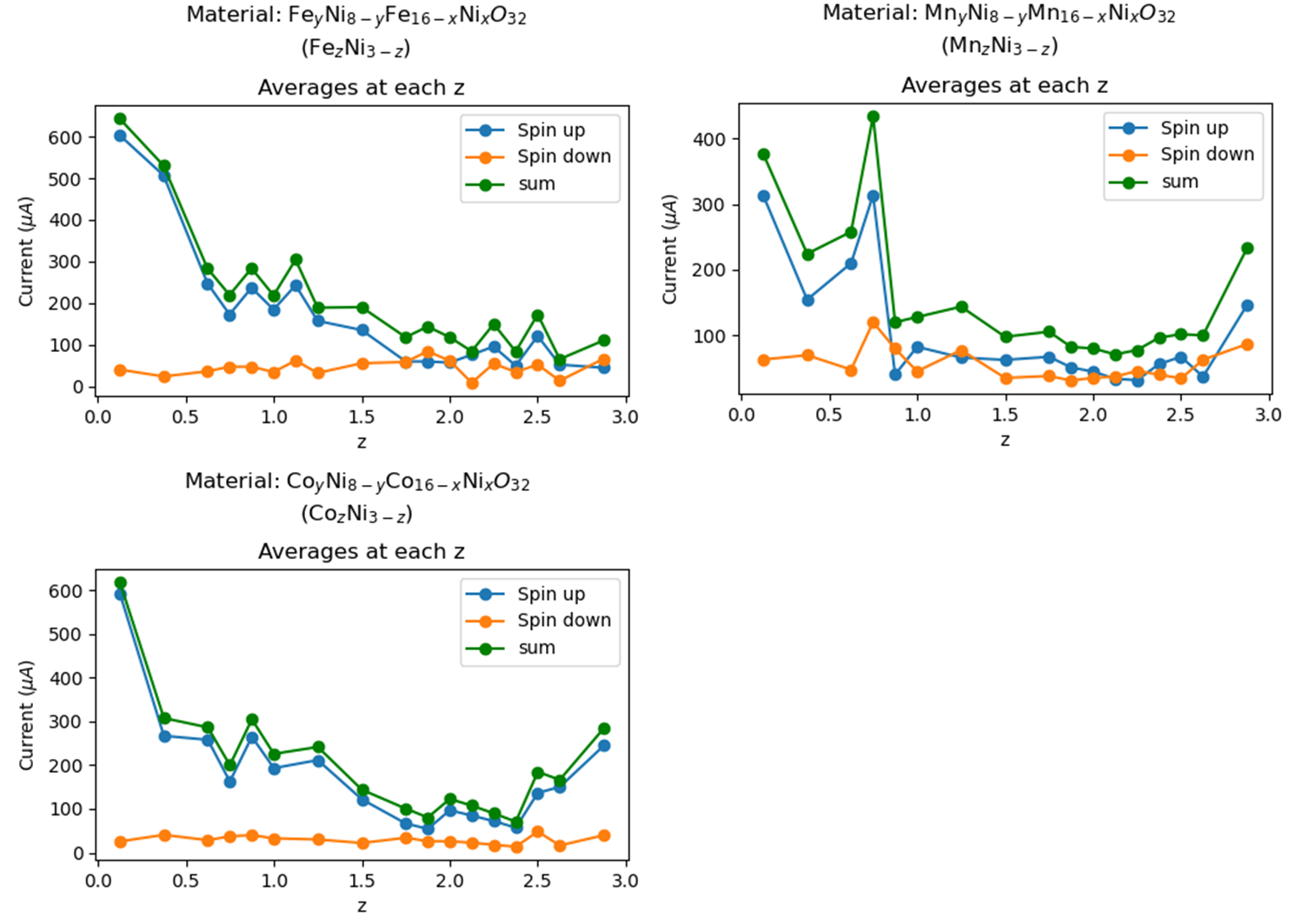


Figure S3. Current calculation results of Co-Ni-O compositions. Top left: all samples results as function of z. Top right: average results per z. Bottom left: results of currents for lowest energy sample at each z composition. Bottom right: full results (red dots) for each sample as function of (x,y) values.


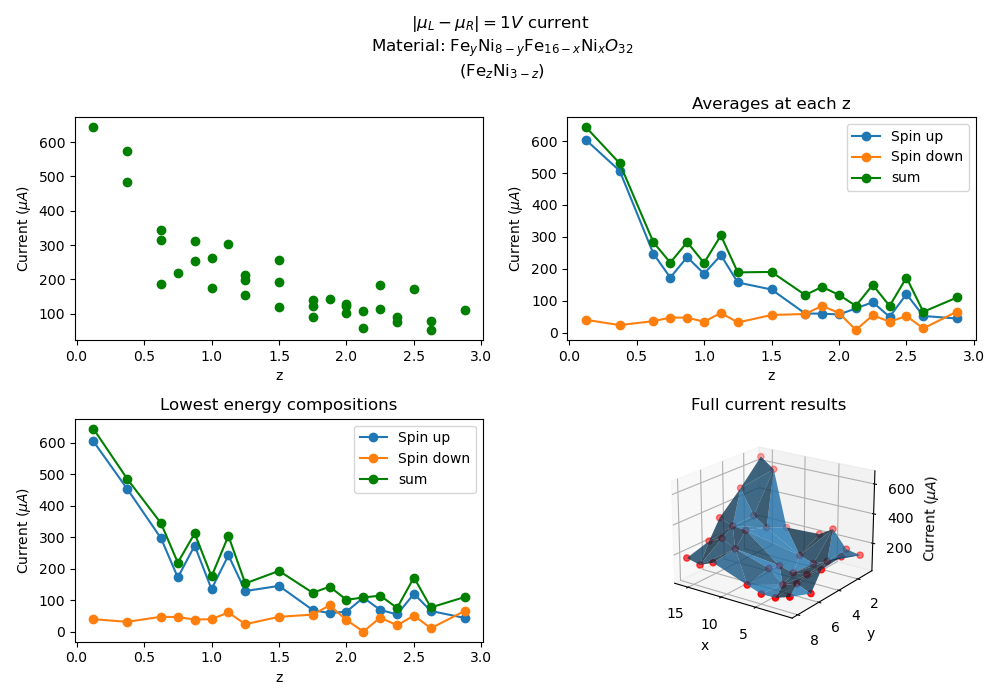


Figure S4. Current calculation results of Fe-Ni-O compositions. Top left: all samples results as a function of z. Top right: average results per z. Bottom left: results of currents for lowest energy sample at each z composition. Bottom right: full results (red dots) for each sample as a function of (x,y) values.


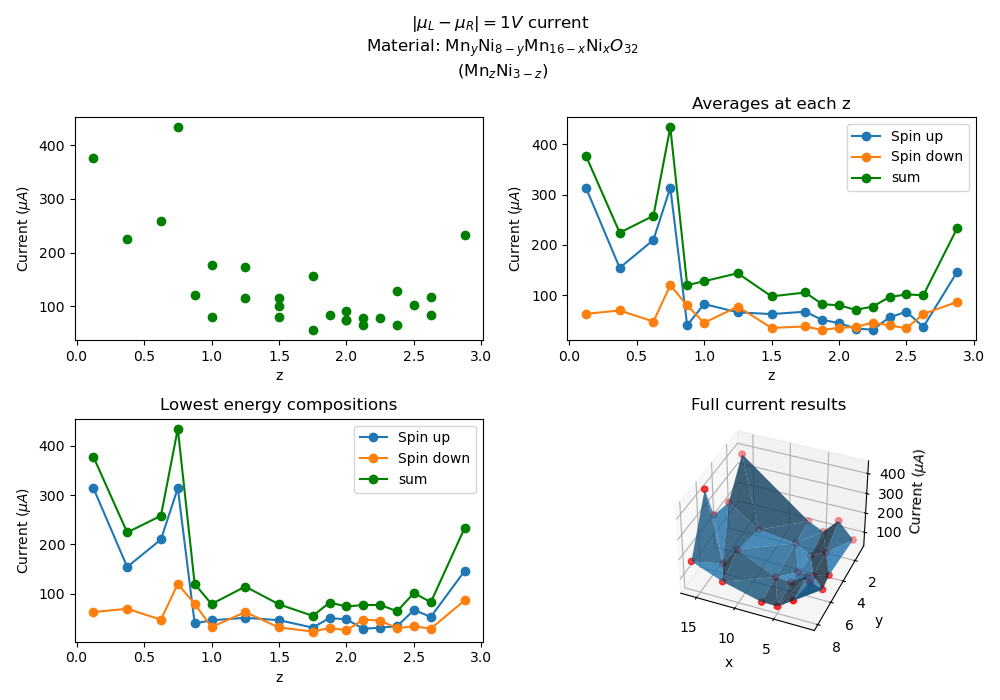


Figure S5. Current calculation results of Mn-Ni-O compositions. Top left: all samples results as function of z. Top right: average results per z. Bottom left: results of currents for lowest energy sample at each z composition. Bottom right: full results (red dots) for each sample as function of (x,y) values.


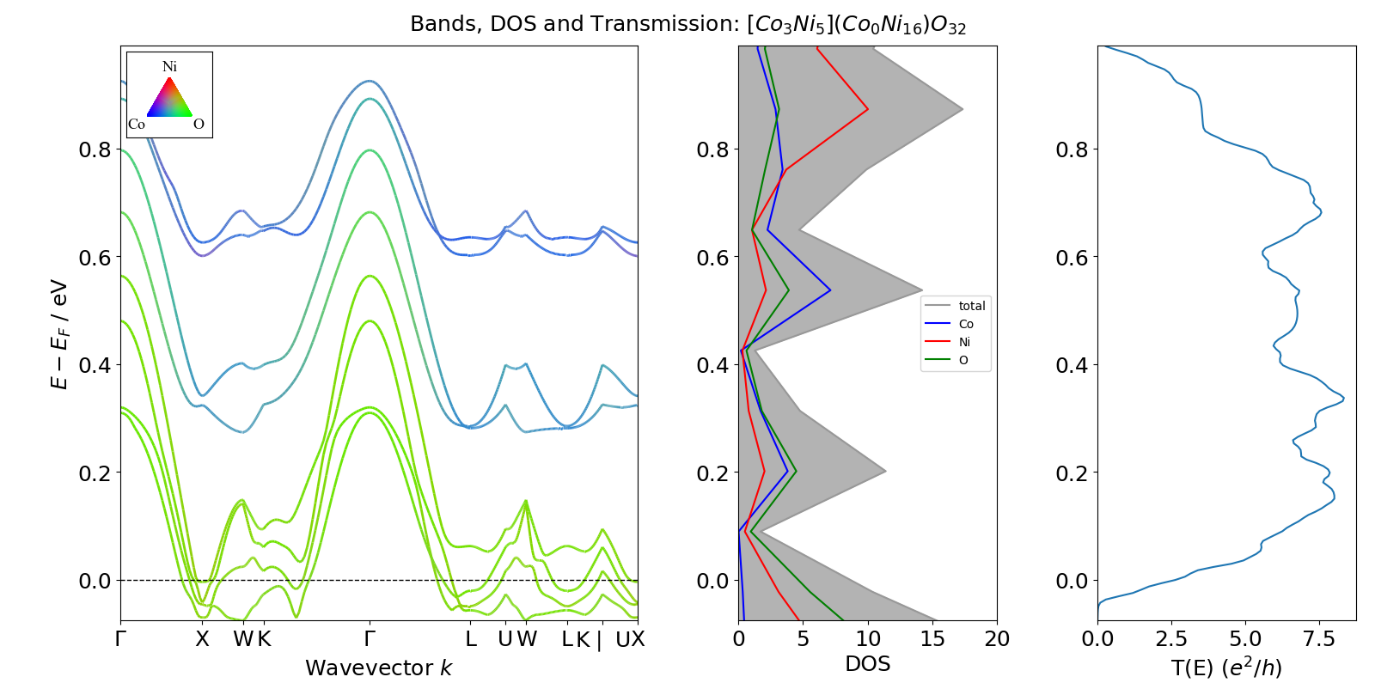


Figure S6. Bands, dos, and transmission results of wide band material Co_3_Ni_21_O_32_.Current at 1V bias is 452µA.


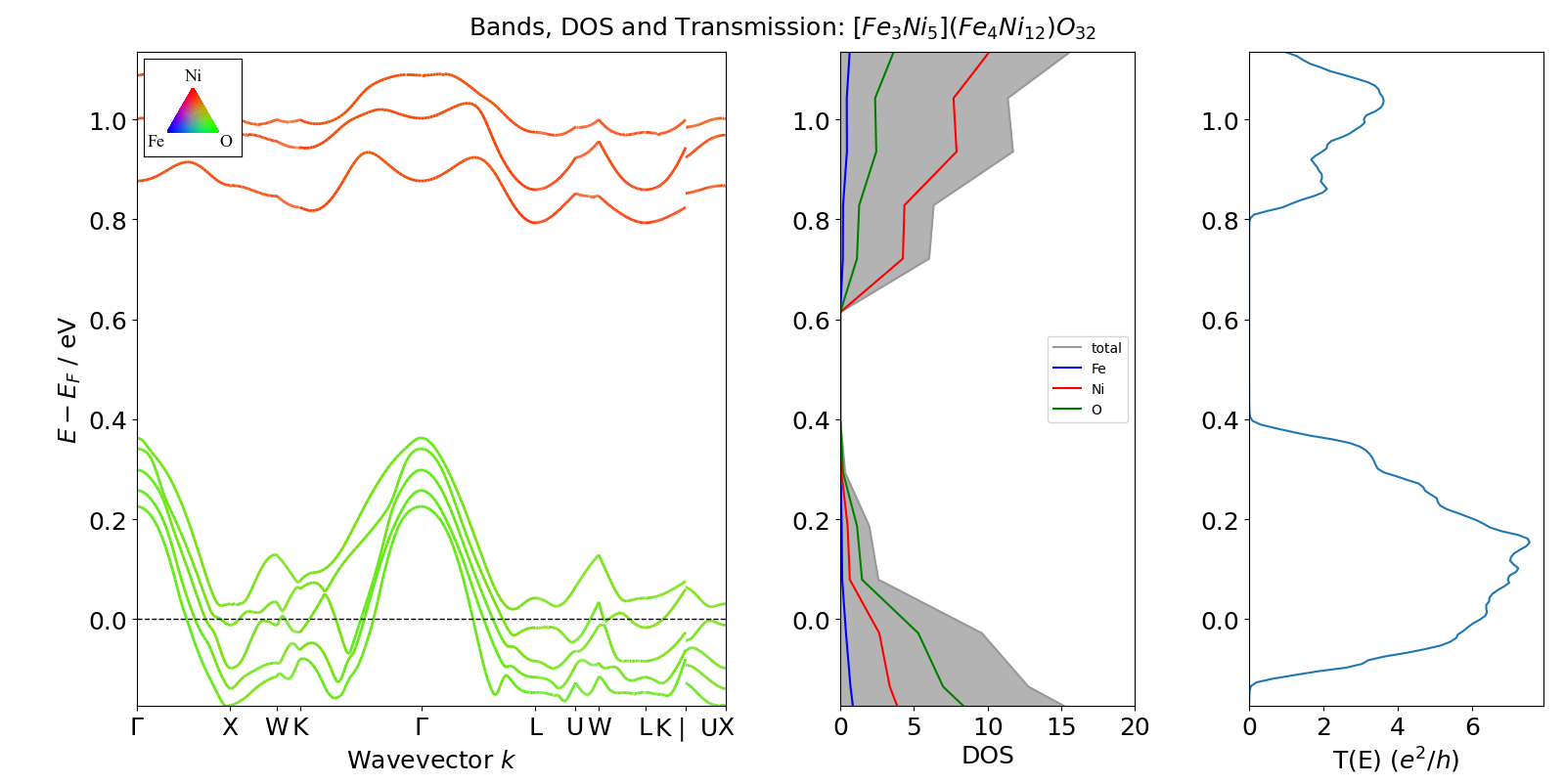


Figure S7. Bands, dos, and transmission results of wide band material Fe_7_Ni_17_O_32_. Current at 1V bias is 252µA.


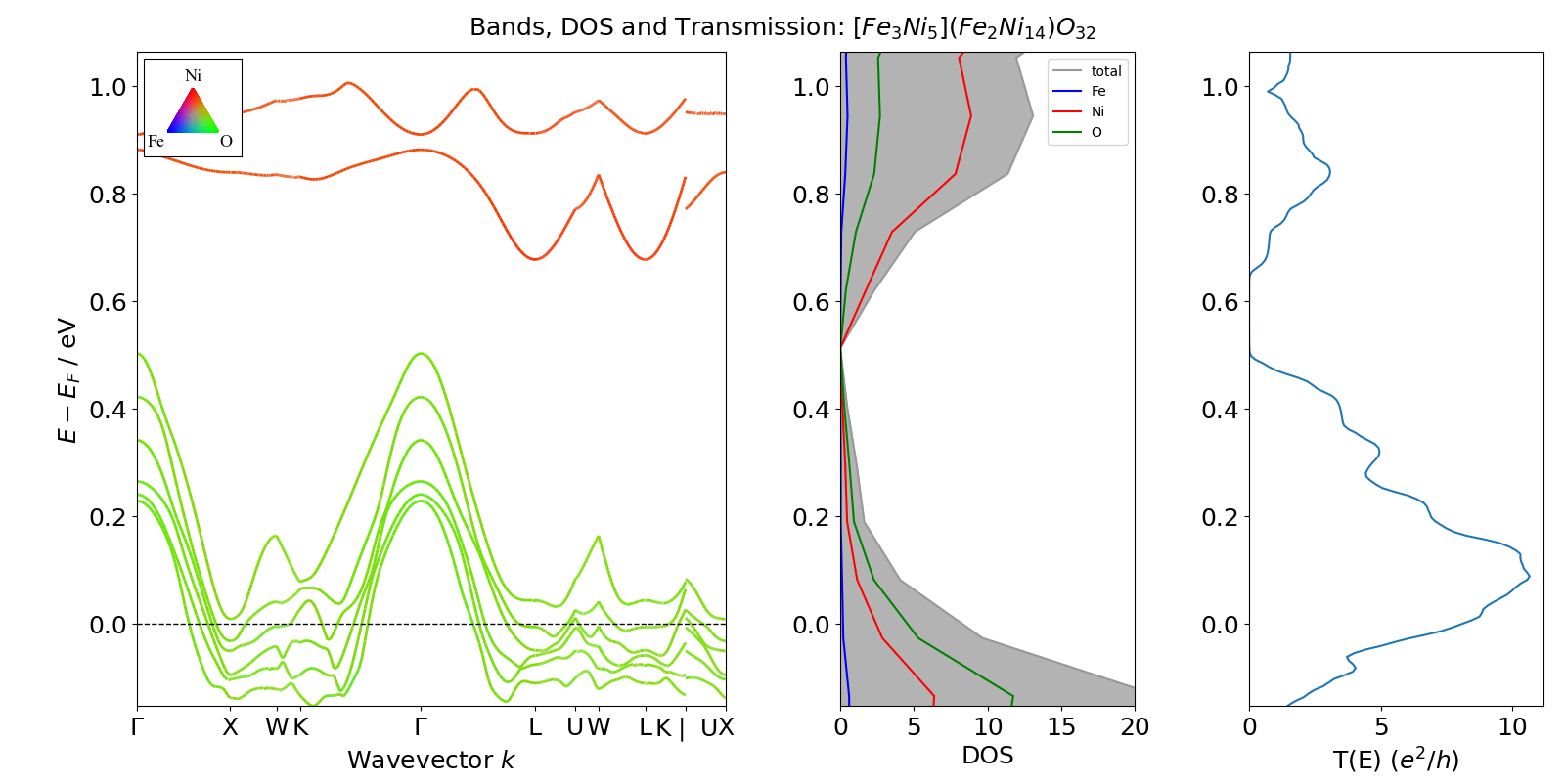


Figure S8. Bands, dos, and transmission results of wide band material Fe_7_Ni_17_O_32_. Current at 1V bias is 329µA.

Table 1. Full classification report of the semiconductor/half-metal classifier.

|  | precision | recall | f1-score | support |
| --- | --- | --- | --- | --- |
| 0 | 0.79 | 0.96 | 0.86 | 23 |
| 1 | 0.90 | 0.60 | 0.72 | 15 |
| accuracy | 0.82 |  |  | 38 |


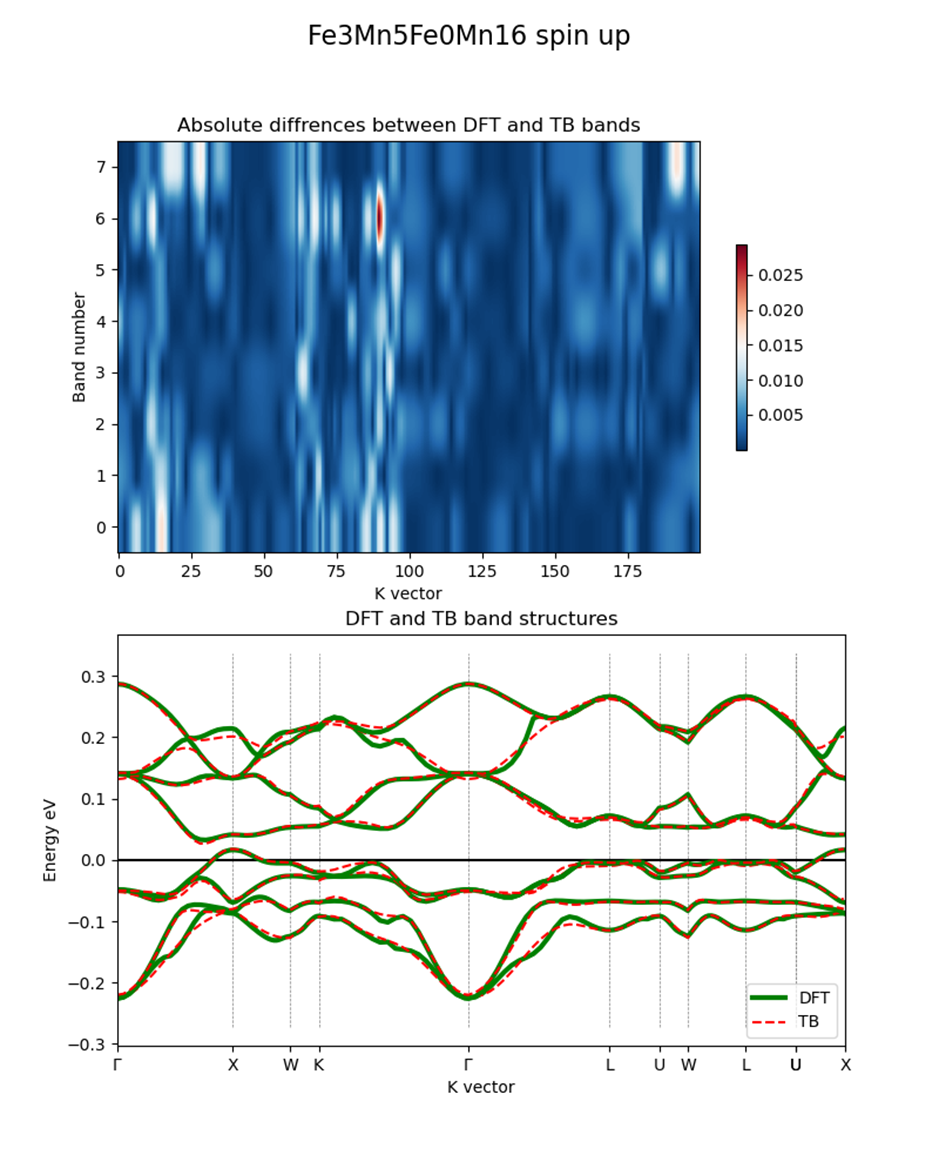

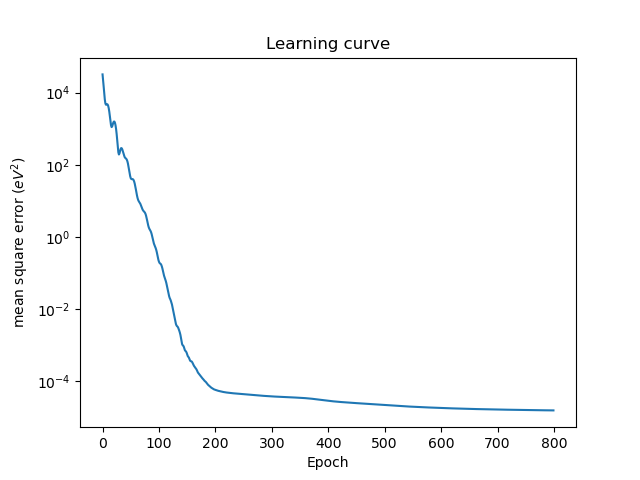


Figure S9. An example of TB fitting results to a spinel sample. The left top plot shows the differences between DFT and TB bands with a color bar. The bottom plot is the bands produced by the fitted tight-binding Hamiltonian (dashed red) on top of the DFT bands(green). The right plot is the learning curve of the fitting. After 700 epochs the band's fitting stays almost the same.

Database

The csv files that sums up the database used to train the models in this work can be found on the supplementary zip file inside the ‘data’ folder.

The files: FC.csv, MC.csv, MF.csv, NC.csv, NF.csv, and NM.csv contains the information about the structures that were used in this work, their bandgaps, and current results. The file names refer to the initials of the elements that included in a specific spinels family.

The file DB_bw_v2.csv contains all the data used to train the bandwidth model.

ML model parameters

*Table S2. Tuned hyperparameters of the composition-current predictor models.*

| **Algorithm** | **Hyperparameters** |
| --- | --- |
| SVR | Epsilon=20  C=100  Gamma=scale  Kernel=rbf |
| KRR | Alpha=0.1  Gamma=1.5  Kernel=rbf |
| RF | max_features=4  n_estimators = 50 |
| NN | Hidden layers=[128,128,128]  Activtation=relu  Optimizer=Adam  Learning rate=1e-2  Weight decay=2.0  Loss=MSE |

*Table S3. Hyper-parameters for bandwidth models.*

| **XGboost hyper-parameters** | **values** |
| --- | --- |
| Number of estimators | 100 |
| eta | 0.043 |
| subsample | 0.8 |
| colsample_bytree | 0.8 |
| max_depth | 12 |
| min_child_weight | 8 |
| reg_alpha | 0.7 |
| reg_lambda | 8.1 |
| **Feed forward neural network hyper-parameters** |  |
| Number of neurons per hidden layer | [512,256,128] |
| Activation function | ReLU |
| Optimizer | Adam |
| Weight decay (L2 regularization) | 1.0 |
| Batch size | 304 (all training set) |

*Table S4. Band gap predictor models hyper-parameters.*

| **Model** | **Parameters** |
| --- | --- |
| XGBoost regression | n_estimators=50  eta=0.5  subsample=0.9  colsample_bytree=0.8,  max_depth=3,  min_child_weight=0.0,  reg_alpha=0.000  reg_lambda=12 |
| XGBoost classifier | n_estimators =300  max_depth=6  reg_alpha=0  reg_lambda=0.1  scale_pos_weight=1  subsample=0.8 |

Code files

The scripts that were used in this work can be found inside the supplementary zip file in the ‘codes’ folder.

General workflow:

Bands.py: a module with different functions to create the band class which is used to hold all necessary data for fitting.

bandsclass_create.py: a script that iterate over all samples and create band class for each one.

TBHfitting.py: a module with different functions to use for bands fitting.

multipleTtrainv4.py: A full TB fitting script that iterates over all compositions in specific folder and saves the results to another folder.

transport_mod_extensionv2.py (the latest version is 2): a module with the class bulk_transport6 for the calculation of NEGF and Landauer formula

BlockTransport.py: a scripts for generating current results from a specific folder that contains different TBHs (pickle files that were saved during fitting process).

Magnetic moments results (before recalculations and fixing anomalies)


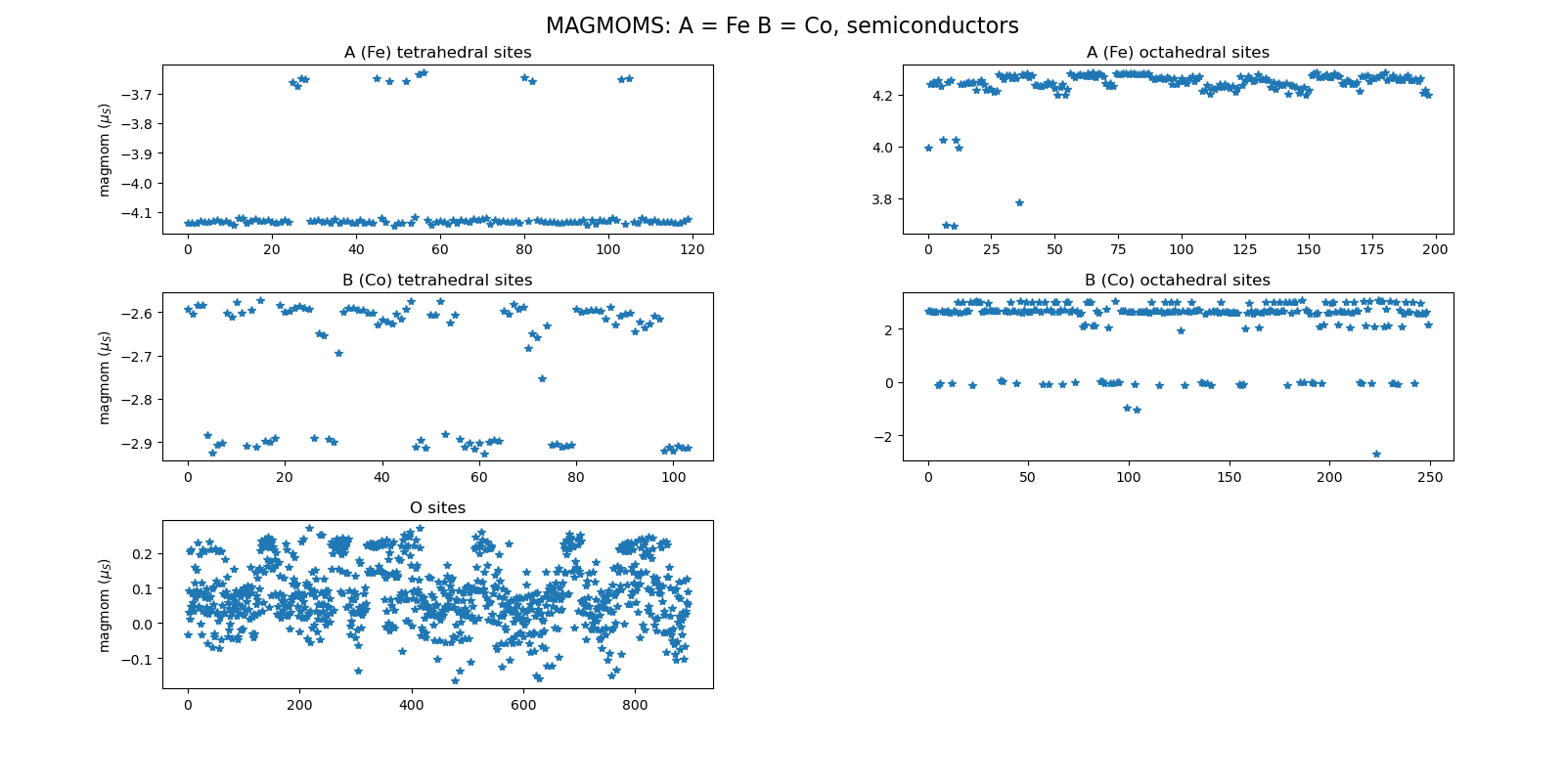


Figure S10. Magnetic moments in Fe-Co-O with band gap system (before re-calculation).


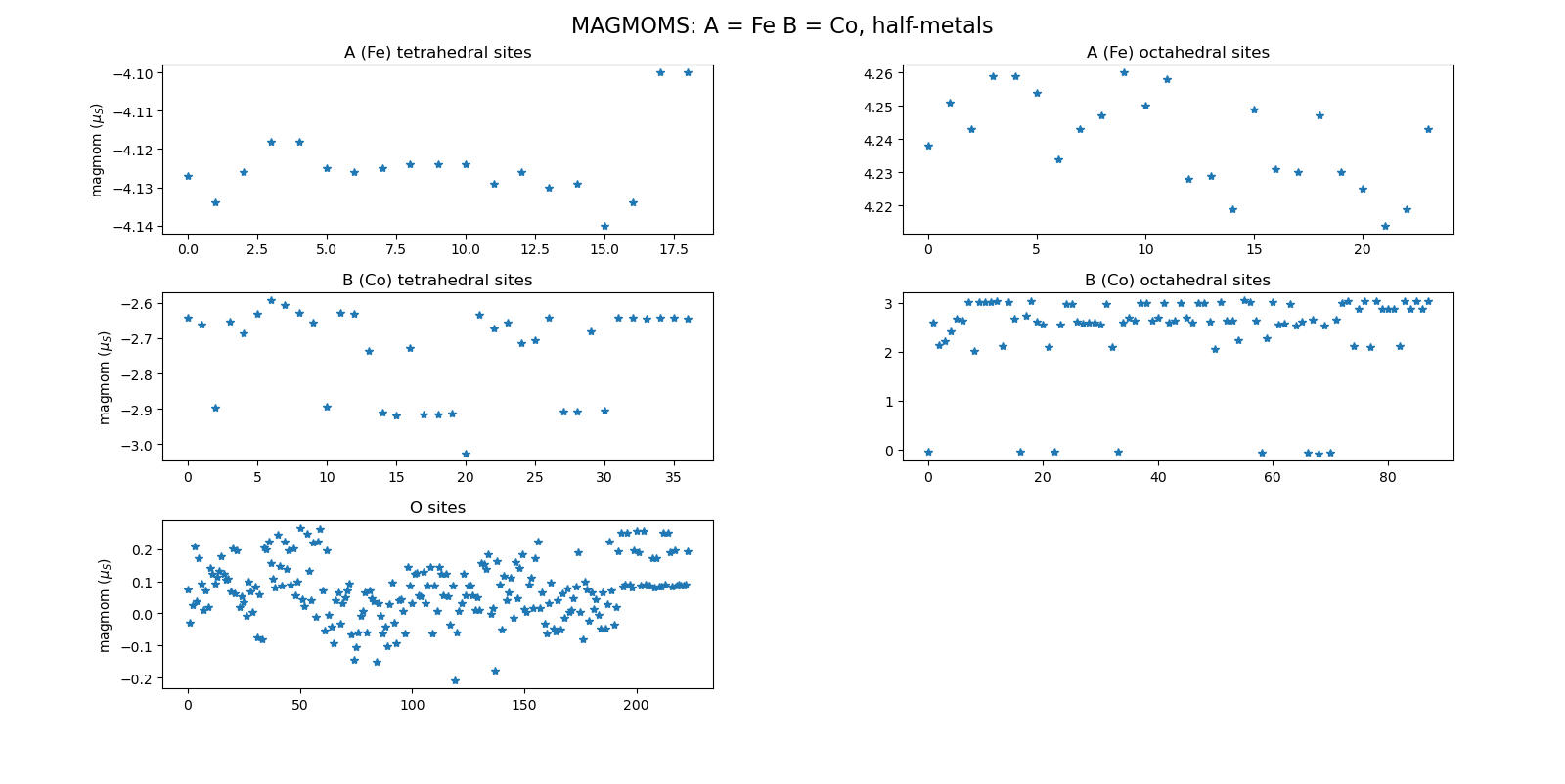


Figure S11. Magnetic moments in Fe-Co-O half-metals system (before re-calculation).


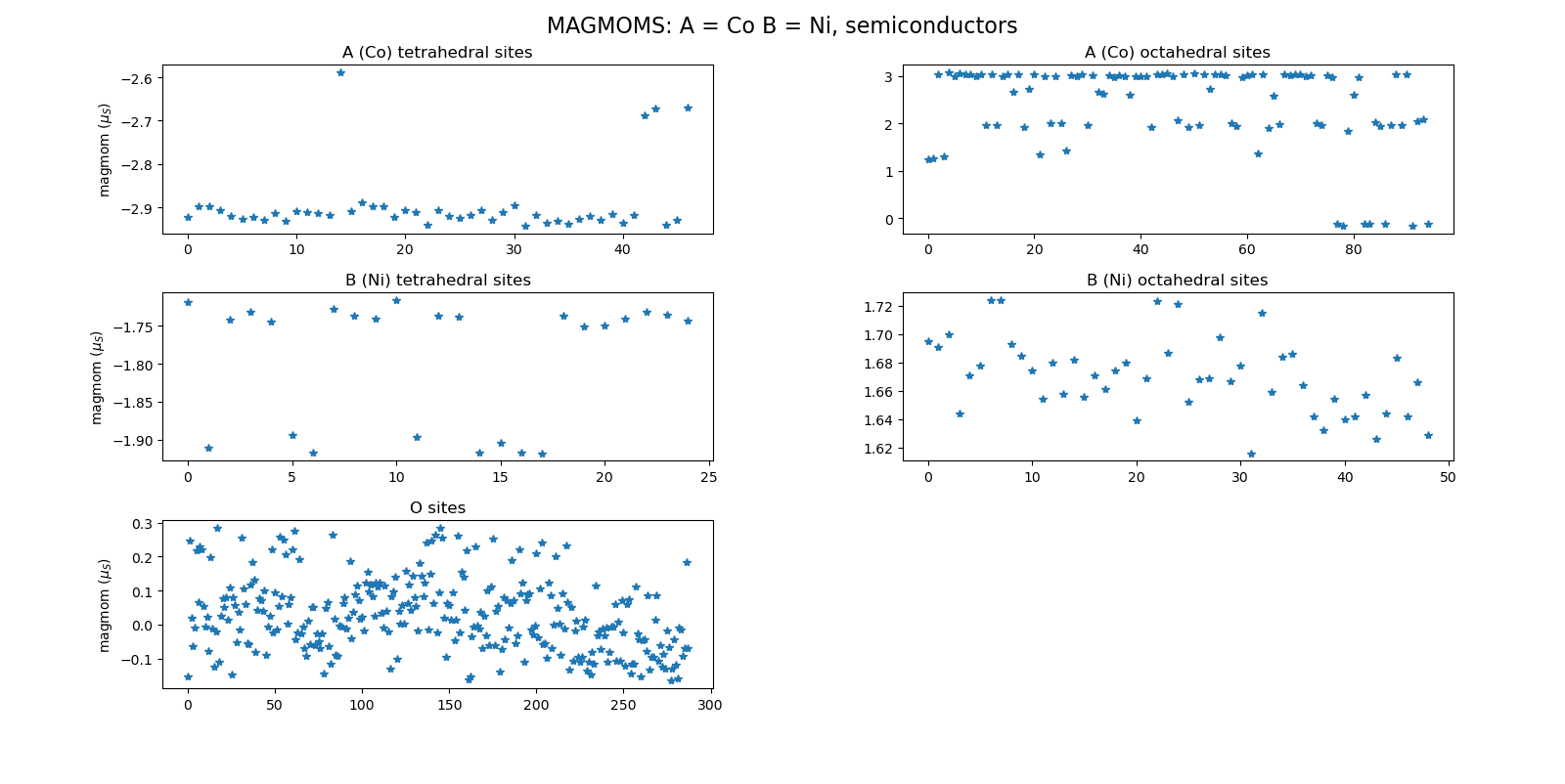


Figure S12. Magnetic moments in Ni-Co-O semiconductor system (before re-calculation).


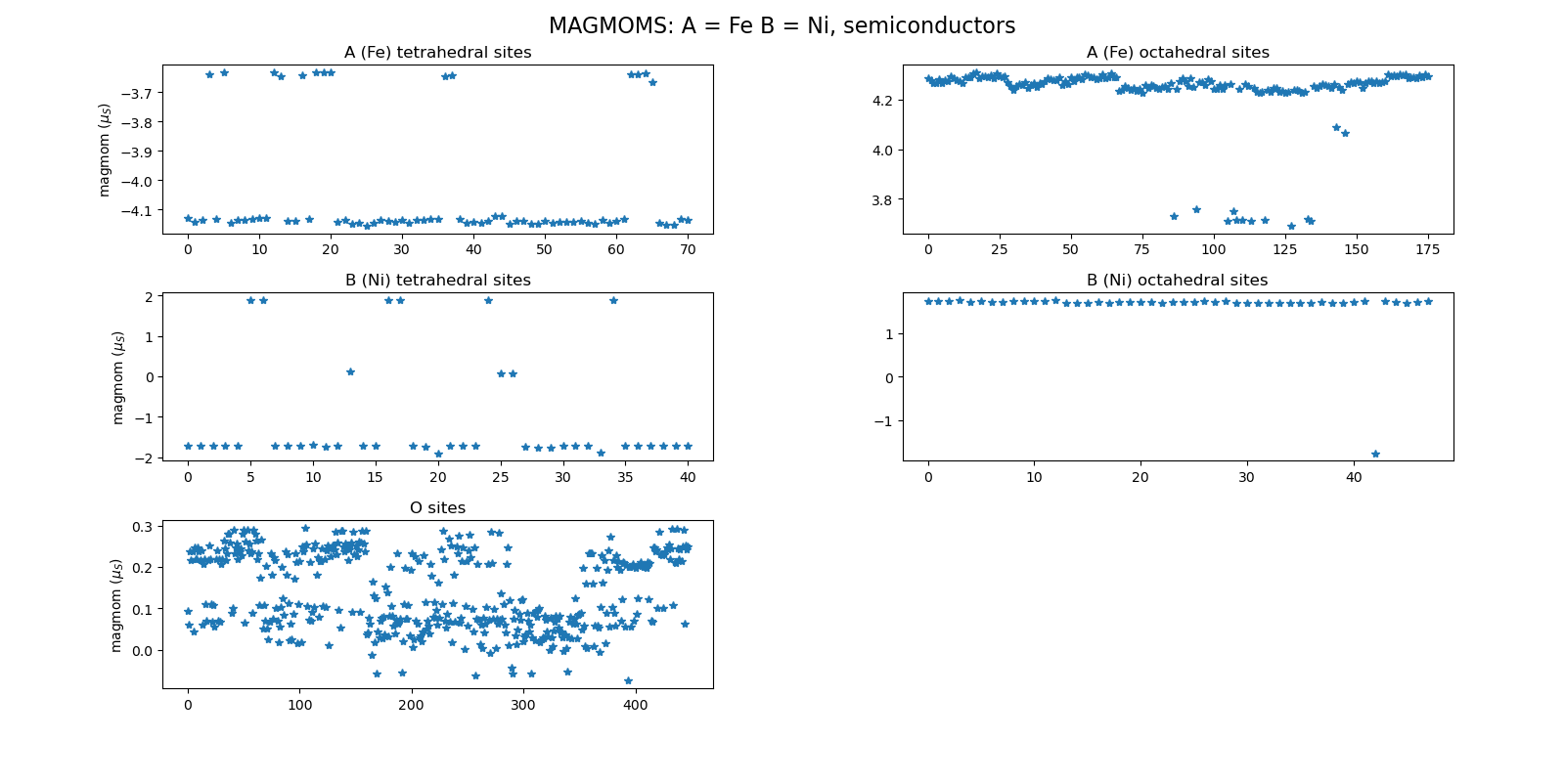


Figure S13. Magnetic moments in Ni-Fe-O semiconductors system (before re-calculation).


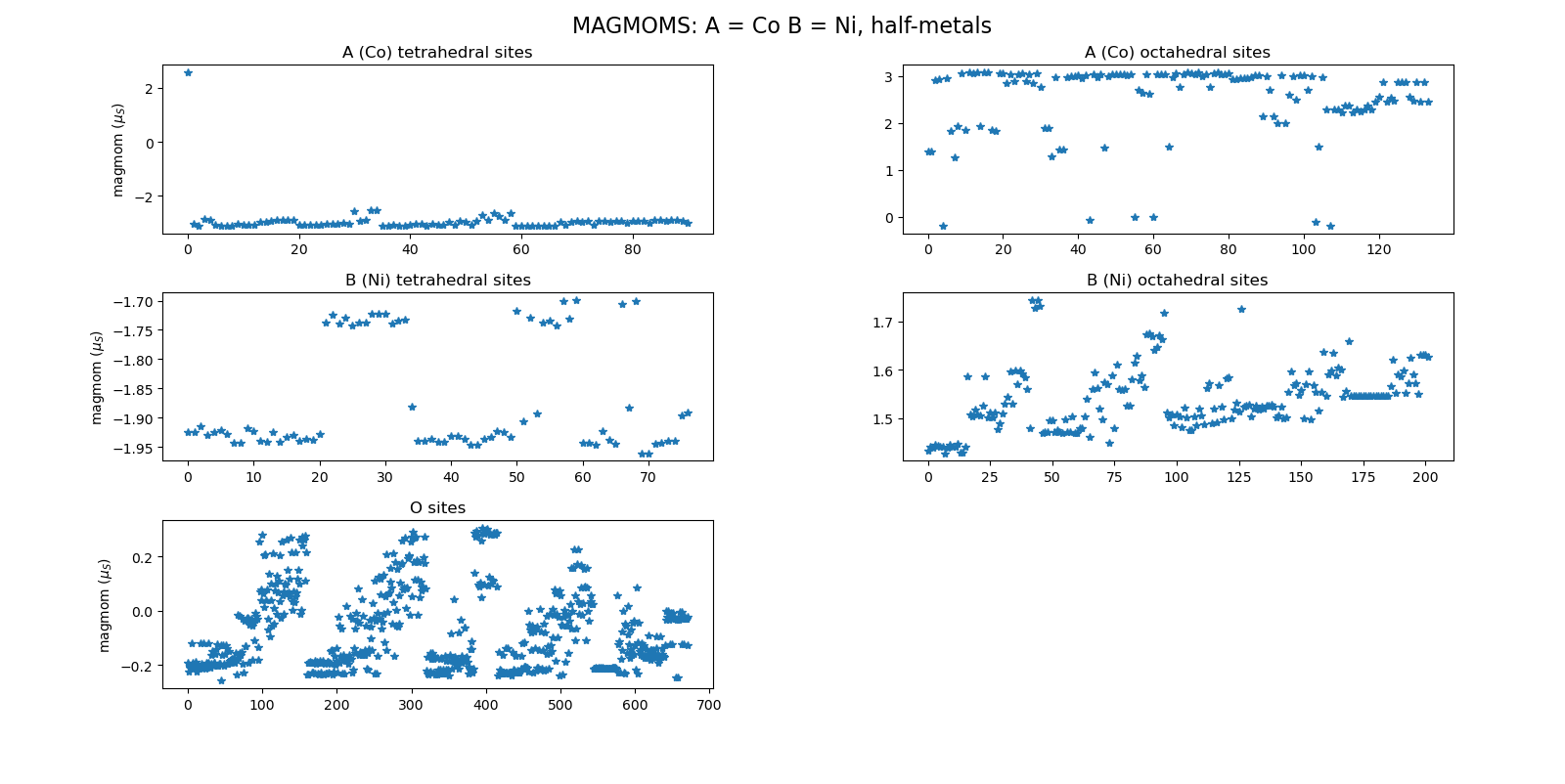


Figure S14. Magnetic moments in Ni-Co-O half-metals system (before re-calculation).


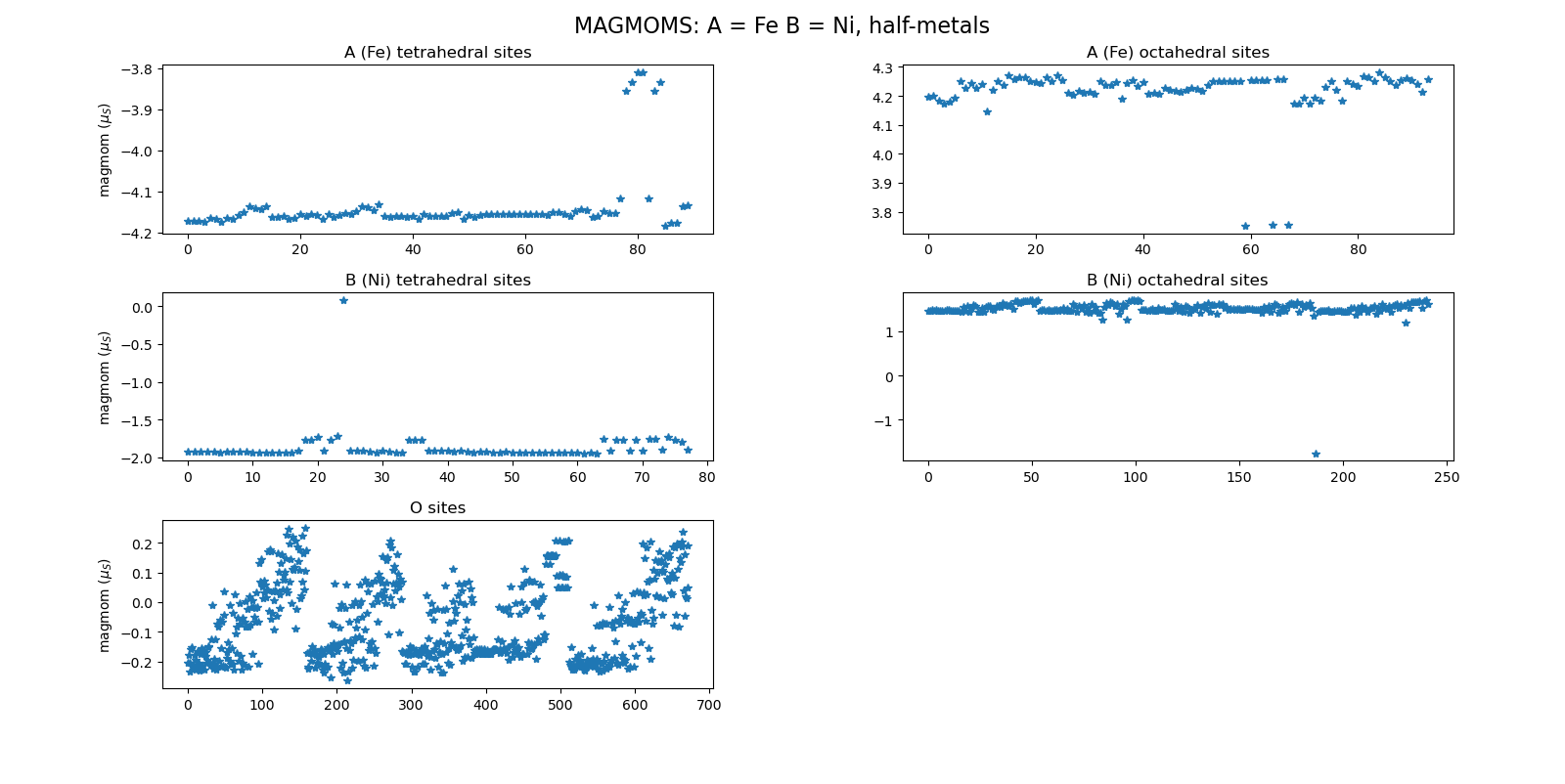


Figure S15. Magnetic moments in Fe-Ni-O half-metals system (before re-calculation).

Band structure k-point file

The following is the KPOINTS file with the high symmetry lines used for band structure calculations:

Line_mode KPOINTS file

40

Line_mode

Reciprocal

0.0 0.0 0.0 ! \Gamma

0.5 0.0 0.5 ! X

0.5 0.0 0.5 ! X

0.5 0.25 0.75 ! W

0.5 0.25 0.75 ! W

0.375 0.375 0.75 ! K

0.375 0.375 0.75 ! K

0.0 0.0 0.0 ! \Gamma

0.0 0.0 0.0 ! \Gamma

0.5 0.5 0.5 ! L

0.5 0.5 0.5 ! L

0.625 0.25 0.625 ! U

0.625 0.25 0.625 ! U

0.5 0.25 0.75 ! W

0.5 0.25 0.75 ! W

0.5 0.5 0.5 ! L

0.5 0.5 0.5 ! L

0.375 0.375 0.75 ! K

0.625 0.25 0.625 ! U

0.5 0.0 0.5 !

Converged structures

All final geometric structures (CONTCAR files) and final energies can be found in the ‘contcars’ directory inside the supplementary zip file.
